# *BUB1B* monoallelic germline variants contribute to prostate cancer predisposition by triggering chromosomal instability

**DOI:** 10.1186/s12929-024-01056-z

**Published:** 2024-07-16

**Authors:** Maria P. Silva, Luísa T. Ferreira, Natércia F. Brás, Lurdes Torres, Andreia Brandão, Manuela Pinheiro, Marta Cardoso, Adriana Resende, Joana Vieira, Carlos Palmeira, Gabriela Martins, Miguel Silva, Carla Pinto, Ana Peixoto, João Silva, Rui Henrique, Sofia Maia, Helder Maiato, Manuel R. Teixeira, Paula Paulo

**Affiliations:** 1 Cancer Genetics Group, IPO Porto Research Center (CI-IPOP) / RISE@CI-IPOP (Health Research Network), Portuguese Oncology Institute of Porto (IPO Porto) / Porto Comprehensive Cancer Center, Porto, Portugal; 2 LAQV, REQUIMTE, Department of Chemistry and Biochemistry, Faculty of Sciences, University of Porto, Porto, Portugal; 3 Department of Laboratory Genetics, Portuguese Oncology Institute of Porto (IPO Porto) / Porto Comprehensive Cancer Center, Porto, Portugal; 4 Department of Immunology, Portuguese Oncology Institute of Porto (IPO Porto) / Porto Comprehensive Cancer Center, Porto, Portugal; 5 Department of Pathology, Portuguese Oncology Institute of Porto (IPO Porto) / Porto Comprehensive Cancer Center, Porto, Portugal; 6 Chromosome Instability & Dynamics Group, Instituto de Investigação e Inovação em Saúde, University of Porto / Porto Comprehensive Cancer Center, Porto, i3S Portugal; 7 Cell Division Group, Experimental Biology Unit, Department of Biomedicine, Faculty of Medicine, University of Porto, Porto, Portugal; 8 Instituto de Biologia Molecular e Celular, University of Porto, Porto, Portugal; 9 School of Medicine and Biomedical Sciences (ICBAS), University of Porto, Porto, Portugal

**Keywords:** *BUB1B*, BubR1, Cancer predisposition, Premature chromatid separation, Chromosomal instability, Taxol response

## Abstract

**Background:**

Prostate cancer (PrCa) is the most frequently diagnosed cancer in men. Variants in known moderate- to high-penetrance genes explain less than 5% of the cases arising at early-onset (< 56 years) and/or with familial aggregation of the disease. Considering that BubR1 is an essential component of the mitotic spindle assembly checkpoint, we hypothesized that monoallelic *BUB1B* variants could be sufficient to fuel chromosomal instability (CIN), potentially triggering (prostate) carcinogenesis.

**Methods:**

To unveil *BUB1B* as a new PrCa predisposing gene, we performed targeted next-generation sequencing in germline DNA from 462 early-onset/familial PrCa patients and 1,416 cancer patients fulfilling criteria for genetic testing for other hereditary cancer syndromes. To explore the *pan*-cancer role of *BUB1B*, we used in silico BubR1 molecular modeling, in vitro gene-editing, and ex vivo patients’ tumors and peripheral blood lymphocytes.

**Results:**

Rare *BUB1B* variants were found in ~ 1.9% of the early-onset/familial PrCa cases and in ~ 0.6% of other cancer patients fulfilling criteria for hereditary disease. We further show that *BUB1B* variants lead to decreased BubR1 expression and/or stability, which promotes increased premature chromatid separation and, consequently, triggers CIN, driving resistance to Taxol-based therapies.

**Conclusions:**

Our study shows that different *BUB1B* variants may uncover a trigger for CIN-driven carcinogenesis, supporting the role of *BUB1B* as a (*pan*)-cancer predisposing gene with potential impact on genetic counseling and treatment decision-making.

**Supplementary Information:**

The online version contains supplementary material available at 10.1186/s12929-024-01056-z.

## Introduction

Prostate cancer (PrCa) is the second most frequently diagnosed cancer and the fifth leading cause of male cancer deaths worldwide [1]. Familial aggregation, Hereditary Breast and Ovarian Cancer Syndrome, and Lynch Syndrome are among the most important risk factors for PrCa development, in addition to age, race, ethnicity, and environmental factors. Nevertheless, little is known about the genetic determinants that underlie PrCa development in patients with early-onset and/or familial aggregation of the disease [hereafter referred to as HPC (hereditary prostate cancer) cases] [2]. In fact, previous screenings in our series of 462 HPC cases revealed that < 5% of the patients are carriers of variants in the DNA damage response or *HOXB13* genes known to be associated with PrCa predisposition [3–5]). As such, new strategies are required to unveil the heterogeneous genetic components underlying hereditary PrCa development.

Aiming to provide new insights into the missing heritability of PrCa, we previously unveiled *CEP57* as a potential PrCa predisposing gene [5]. Biallelic deleterious variants in *CEP57* were described in 2011 as a new cause of Mosaic Variegated Aneuploidy (MVA), leading to the classification of the MVA2 syndrome, which contrasts with the MVA1 syndrome, caused by biallelic variants in the *BUB1B* gene. Although aneuploidy is a known cancer hallmark, there are no reports of cancer development in MVA2 patients, with only thirteen cases reported to date [6]. For MVA1 syndrome, the frequency of cancers (mostly in childhood) is not higher than 40% [7], eventually biased by the high rates of early mortality. Although scarce, carriers of *BUB1B* heterozygous deleterious variants have been reported in some cancer patient cohorts, namely, early-onset colorectal cancer [8], familial pancreatic cancer [9], and cutaneous melanoma with multiple primary carcinomas [10]. Nevertheless, the role of *BUB1B* as a cancer-predisposing gene is not yet clarified.

BubR1 (Budding uninhibited by benzimidazole-related 1), encoded by the *BUB1B* gene, is an essential component of the Spindle Assembly Checkpoint (SAC), a surveillance mechanism that prevents persistent chromosome missegregation (defined as Chromosomal Instability or CIN) and aneuploidy [11, 12]. SAC function relies on the proper assembly of a mitotic checkpoint complex (MCC) catalyzed at unattached kinetochores (KTs). The MCC is composed of Mad2, Bub3, BubR1, and Cdc20, and acts by inhibiting APC/C (anaphase promoting complex/cyclosome) activation, which is essential for mitotic exit [13]. Several studies have shown that partial reduction of the MCC is sufficient to severely impair SAC signaling leading to chromosome segregation fidelity defects, aneuploidy and CIN, hallmarks of cancer [14, 15]. MVA1 syndrome and the Premature Chromatid Separation (PCS) trait, caused by biallelic and monoallelic *BUB1B* variants, respectively, are the strongest evidence for a causal effect of CIN in cancer development arising from SAC defects [16–19]. However, solid and mechanistic evidence establishing a link between specific *BUB1B* monoallelic variants and cancer predisposition are lacking. Additionally, considering the association between CIN levels and the response to taxane-based chemotherapeutic drugs [20, 21], the identification of carriers of deleterious *BUB1B* variants may also have significant implications for treatment decision-making.

In this work, we aimed to assess the frequency of carriers of germline variants in *BUB1B* among patients with criteria for hereditary PrCa and to clarify the biological mechanisms by which *BUB1B* variants might trigger carcinogenesis in human cells, as well as to evaluate their possible therapeutic implications. By combining in vitro, ex vivo and in silico approaches, we show that rare *BUB1B* variants are prevalent in early-onset/familial PrCa and in other cancer syndromes, and trigger oncogenic transformation by destabilizing BubR1, leading to PCS and CIN, and, consequently, taxane-resistant proliferation. Altogether, our results provide novel insights into the underlying mechanisms of (prostate) carcinogenesis and highlight *BUB1B gene* in the roadmap of genetic counselling and precision medicine.

## Materials and methods

### HPC samples

This study used germline DNA from 462 index cases of early-onset/familial PrCa (HPC) cases, previously recruited [4]. The patients were recruited based on two criteria: early-onset disease, with PrCa diagnosis before the age of 56, and/or familial/hereditary PrCa, with more than one case with PrCa and at least one of them diagnosed before the age of 66. Of the 462 HPC cases, 240 (51.9%) fulfilled the early-onset disease criterion, and 311 (67.3%) fulfilled the family history criterion, with 89 (19.3%) fulfilling both criteria. Demographic and clinicopathological characteristics of all the carriers are listed in Table S1.

### Targeted next-generation sequencing (T-NGS)

We used a customized gene panel designed with Agilent SureDesign (Agilent Technologies, Santa Clara, CA, USA) to sequence germline DNA from the index patients of all 462 PrCa cases. The panel covered, among other PrCa genes under investigation, the coding and splicing regions of *BUB1B* (NM_001211.5). Capture and sequencing, data processing, variant annotation, and prioritization were performed as previously described [22]. Detailed information is described in Supplementary Methods.

### Dataset of patients tested for other hereditary cancer syndromes

To gain insights into the possible *pan*-cancer role of pathogenic/likely pathogenic variants in *BUB1B*, we searched for *BUB1B* carriers among 1,416 cancer patients referred for molecular diagnosis of multiple inherited cancer syndromes at the Department of Genetics of IPO Porto, already screened with the TruSight Cancer Panel v.1 (Illumina). Variant annotation and filtering were performed as described for the custom T-NGS panel mentioned above and detailed in Supplementary Methods.

### Control samples

To estimate the risk between the carrier status for the c.1171_1173del variant and PrCa development, we used germline DNA from 459 healthy male individuals (mean age 48.3 years; SD ± 10.2 years) as control samples, including 288 blood donors from the Portuguese Oncology Institute of Porto with no personal history of cancer at the time of blood collection and 171 healthy relatives with negative predictive genetic testing (each from independent families). To estimate the global risk for cancer development, germline DNA from 416 healthy females (mean age 48.6 years; SD ± 9.7 years) was also used, which included 243 blood donors and 173 healthy relatives with negative predictive genetic testing.

To assess the basal levels of the PCS trait in our population, we performed PCS on normal lymphocyte metaphase spreads of ten age-matched healthy males aged between 42 and 78 years (mean age 52.6 years; SD ± 10.7 years), specifically recruited for this purpose, under signed informed consent.

### KASP genotyping

Kompetitive Allele Specific PCR (KASP) genotyping, with variant-specific KASP probes, was performed according to the manufacturer’s instructions. Assay primers (Metabion, Köln, Germany) summarized in Table S2 were designed using the Primer-BLAST design tool from the National Center for Biotechnology Information (NCBI) [23], and the PCRs were run on a LightCycler 480 Real-Time instrument (Roche Life Sciences, Basel, Switzerland). For data analysis, LightCycler 480 Software 1.5.0 was used.

### Haplotype analyses

The T-NGS data were phased using BEAGLE 4.1 [24], and IBD haplotypes were determined using the BEAGLE Refined IBD algorithm [25]. The lengths of the shared haplotype segments were estimated by the distance between the last two shared markers flanking the variants. A similar IBD and haplotype approach was applied to the high-density SNP genotype data from the Portuguese early-onset/familial PrCa sample collection (374 PrCa cases and 180 controls) obtained with the Infinium OncoArray-500 K BeadChip (Illumina) as part of the PRACTICAL consortium, as previously described [26].

Microsatellite haplotype analysis was performed using nine polymorphic microsatellite markers flanking the gene, namely, D15S118, D15S1012, D15S1044, D15S146, D15S214, TR20GT, D15S968, AFM196XB8, and D15S781. A total of seven probands carrying the *BUB1B* c.1171_1173del variant were genotyped, including all five HPC patients, the two additional carrier patients found among the 1,416 screened for multiple hereditary cancer syndromes (Table 2), and seven unaffected family members. Primers were designed using the Primer-BLAST tool [23] (Table S2) and acquired from Metabion. All markers were assayed by PCR using fluorescently end-labeled primers, and PCR products were run on a 3500 Genetic Analyzer together with the fluorescence-labeled DNA fragment size standard 600-LIZ (Thermo Fisher Scientific, Waltham, MA, USA). Haplotype construction was performed manually based on the genotypes obtained from probands and family members.

### Next-generation DNA and RNA sequencing of FFPE tumor samples

To assess the impact of *BUB1B* germline variants on *BUB1B* mRNA expression and transcriptomic profiles in the corresponding prostate carcinomas, available prostate tissue samples from carriers were submitted to RNA sequencing. For this purpose, matched tumor and normal RNA were extracted from ~ 5 μm sections of formalin-fixed paraffin-embedded (FFPE) prostate tissues after deparaffinization using xylene and ethanol, according to the recommendations of the High Pure FFPET RNA Isolation Kit (Roche Life Sciences). For each library preparation, 100 ng of total RNA was used as input for the TruSeq™ RNA Exome (Illumina) protocol, according to the manufacturer’s instructions. Libraries were sequenced on an Illumina NextSeq 500 using the NextSeq High Output v2.5 kit using the pair-end run mode with a run setup of 2 × 76 bps. Data was processed for both transcript expression and variant calling, as described in Supplementary Methods. 

To look for a somatic “second hit” in *BUB1B* in the tumors of HPC patients carrying germline variants, DNA was extracted from ~ 5 μm sections of FFPE tumor tissues using the Cobas® DNA Sample Preparation Kit (Roche Life Sciences) after deparaffinization with xylene and ethanol, according to the manufacturer’s recommendations. A T-NGS custom panel covering, among other genes, the coding and splicing regions of *BUB1B* (NM_001211.5), designed with Agilent SureDesign, was used. Capture, sequencing, data processing, and variant annotation were performed as described for germline DNA.

### Generation of prostate in vitro cell models carrying the *BUB1B* variant c.1171_1173del

The human non-tumorigenic prostate-derived cells RWPE-1 were kindly provided by Prof. Margarida Fardilha (University of Aveiro, Portugal). Wild-type RWPE-1 and derived monoclonal cell populations were cultured in sterile conditions, at 37ºC, 5% CO_2_, and a humidified atmosphere, in Kerotinocyte Serum-Free (KSF) Medium (Gibco) supplemented with L-glutamine, 0.005 ng/µL Human Recombinant Epidermal Growth Factor (EGF), and 50 ng/µL of Bovine Pituitary Extract (BPE), and 1% penicillin–streptomycin (Gibco).

To induce specific editing of the *BUB1B* gene in RWPE-1 cells, we used the Alt-R CRISPR/Cas9 System from Integrated DNA Technologies (IDT, Coralville, IA, USA) following the manufacturer’s instructions. Among 153 isolated clones, gene-editing leading to the presence of the recurrent *BUB1B* variant c.1171_1173del was observed in four independent clones, in which gene-editing also led to the occurrence of a second in-frame variant (c.1133_1156del) in the other allele, predicted to lead to loss of amino acids from Proline 378 to Histidine 385 [p.(Pro378_His385del)]. No heterozygous clones with monoallelic gene-editing for the c.1171_1173del variant were found. Thus, we randomly selected two of the four clonal populations with the same genotype (BubR1^Δ391/Δ378−385^) – named C1 and C2 – for further phenotypic evaluation. Detailed information is described in Supplementary Methods.

### Sanger sequencing

For Sanger sequencing, monoclonal cell populations were lysed directly in the well of a 96-well plate with 10 µL of reaction buffer mixture of the Xpert directXtract Lysis Buffer (GRiSP, Porto, Portugal) after a D-PBS wash (Gibco, Thermo Fisher Scientific). Cell lysates were incubated at 75ºC for 5 min, followed by heat-inactivation at 95ºC for 10 min. The cell lysate was diluted 5 × for PCR amplification of the *BUB1B* in-frame deletion, as previously described [5]. Specific primers were designed using Primer-BLAST [23] (Table S2) and acquired from Metabion. The PCR products were purified with Exo/SAP Go (GRiSP) and forwarded for sequencing PCR using the BigDye Terminator v3.1 Cycle Sequencing Kit (Thermo Fisher Scientific), according to the manufacturers’ instructions. Samples were run in a 3500 Genetic Analyzer (Thermo Fisher Scientific).

### Western blot

To assess steady-state BubR1 expression levels, total protein extracts were obtained from 90% confluent cells. Briefly, adherent cells were washed twice with ice-cold D-PBS and scrapped with ice-cold lysis buffer (50 mM Tris HCl, pH 7.5, 150 mM NaCl, and 0.1% NP-40). After incubation on ice for 15 min, soluble fractions were collected by centrifugation at 13,000 rcf for 30 min.

To obtain mitotic extracts, 90% confluent cells in T75 flasks were synchronized with 0.5 µM nocodazole. Mitotic cells were harvested by shake-off and centrifugation at 1200 rpm for 5 min. Cell pellets were washed once with D-PBS and resuspended in ice-cold lysis buffer (50 mM Tris HCl, pH 7.4, 150 mM NaCl, 1 mM EDTA, 1 mM EGTA, 0.5% NP-40, and 0.5% Triton X-100) supplemented with a cocktail of protease inhibitors (Roche Life Sciences). Proteins (50 µg) were separated in a 10% SDS-PAGE system and transferred to a nitrocellulose membrane using the Trans-Blot Turbo Transfer System (Bio-Rad Laboratories, Hercules, CA, USA). BubR1, Bub3, Cdc20 and β-actin were probed using the following antibodies: rabbit anti-BubR1 (1:1,000; ab70544, Abcam, Cambridge, UK), anti-Bub3 (1:2,000; 27,073–1-AP, Proteintech, Rosemont, IL, USA), mouse anti-cdc20 (1:250; sc-5296, Santa Cruz Biotechnology, Santa Cruz, CA, USA), and anti-β-actin antibody (1:10,000; A1978, Sigma-Aldrich). Anti-rabbit (1:10 000) and anti-mouse (1:2,500) HRP-conjugated secondary antibodies (Sigma-Aldrich and Bio-Rad Laboratories, respectively) were visualized using Clarity Western ECL Substrate (Bio-Rad Laboratories).

### Cycloheximide (CHX) chase assay

Cells at 80–90% confluence in T25 flasks were treated with 20ug/mL of cycloheximide (Sigma-Aldrich; C4859) without medium change. At different time points (0 h, 4 h, 8 h and 16 h), total protein extracts were obtained and analyzed by western blot, as described above. Two independent experiments were performed for each clonal cell line paired with WT cells. Images were acquired in the ChemiDoc™ XRS System and data quantified in ImageJ.

### Quantitative PCR (qPCR)

To evaluate the expression levels of *BUB1B* in the gene-edited cell line models and WT cells, total RNA was extracted from each cell population using the RNEasy Mini Kit (QIAGEN, Hilden, Germany), and cDNA was synthesized using the H-minus RevertAid cDNA synthesis kit (Fermentas, part of Thermo Fisher Scientific) with oligo-dT primers, according to the manufacturers’ protocols. Primers and probes (TaqMan) for *BUB1B* were designed using Primer3 software (v4.1, https://primer3.ut.ee) and acquired from Metabion (Martinsried, Germany) (Table S2). The beta-glucuronidase (*GUSB*) housekeeping gene was used as an endogenous control for normalization of the expression levels, and the primers/probe mix was acquired as a pre-developed TaqMan Gene Expression Assay from Applied Biosystems (part of Thermo Fisher Scientific). Relative expression levels were obtained by calibrating *GUSB* normalized *BUB1B* expression values from each population for the expression levels of the WT control population.

### Proliferation and apoptosis assays

Cellular proliferation/viability and apoptosis levels were evaluated with MTT (Sigma-Aldrich) and APOPercentage (Biocolor, Carrickfergus, UK) colorimetric assays, respectively, as previously described [27, 28]. Growth rate (GR) was calculated from the absorbance values obtained with the MTT assay using the formula GR = (T96-T0)/T0. To evaluate Taxol growth inhibition, complete growth medium containing 10 nM Taxol or an equivalent volume of the drug vehicle (DMSO) was added to cells at T0 (h), and the MTT assay was evaluated at T96 (h). For each cell population, the percentage of growth inhibition was calculated using the formula % Inhibition = 100x [1- (T96-T0)_Taxol_/(T96-T0)_Vehicle_], and the percentage of apoptosis was obtained by correcting apoptotic cells to the “total cells” (sum of viable and apoptotic cells) at T96 (h). Statistical data was obtained from four independent experiments.

### Time-lapse microscopy

Live cell analysis was performed under conditions of mitotic arrest generated by persistent unattached KTs using 0.5 µM nocodazole (Sigma-Aldrich) for 16 h. Mitotic controls and *BUB1B*-edited cell clones were imaged by phase-contrast microscopy every 20 min for 36 h using an IN Cell Analyzer 2000 microscope (GE Healthcare, CH, IL, USA) at 37 °C in complete KSF medium. Image processing and quantification were performed in ImageJ.

### Premature Chromatid Separation (PCS) analysis

Peripheral blood samples were cultured for 72 h in RPMI 1640 medium with GlutaMAX-I (Gibco) supplemented with 20% fetal bovine serum (Gibco) and stimulated with Phytohaemagglutinin-M (Biological Industries, Israel). Colcemid™ (100 ng/mL, Gibco) was added 90 min before cell harvesting by trypsinization and cells were processed for G-banding with Leishman staining, according to standard protocols. The analysis of PCS in patients HPC343 and HPC369 carrying missense variants was not performed due to the absence of biological material.

For analysis of PCS levels in cell lines, cells at 90% confluence in T75 flasks were Colcemid™-synchronized for 16 h before harvesting and G-banding processing.

Automatic capture of metaphases from each case was performed using the microscope slide scanning system GSL-120 (CytoVision version 7.4; Leica Biosystems, Baden-Wurttemberg, Germany). The percentage of metaphases with PCS, corresponding to cells with separate and splayed chromatids with discernible centromeres, involving all or most chromosomes of a metaphase cell, was quantified manually by a certified cytogeneticist.

### High-throughput screening of lagging chromosomes

Quantification of lagging chromosomes was performed using 50–500 images of contiguous fields acquired in an IN Cell Analyzer 2000 microscope (GE Healthcare, CH, IL, USA) with a Nikon 40 × /0.95 NA Plan Fluor objective (binning 2 × 2) using a large chip CCD Camera (CoolSNAP K4) with a pixel array of 1,024 × 1,024 (2.7027 pixel/µm resolution), as previously described [29]. All early to late anaphase figures were classified regarding the presence or absence of lagging chromosomes. Any DAPI-positive material between the two chromosome masses, but distinguishably separated from them, was counted as lagging chromosomes. DNA bridges were excluded from this analysis.

### Immunofluorescence

RWPE-1 cells were fixed with 4% paraformaldehyde in PBS (Electron Microscopy Sciences) for 10 min and permeabilized with 0.5% Triton X-100 (Sigma-Aldrich) for another 5 min. BubR1, Cdc20 and ACA were immunostained using the following antibodies: rabbit anti-BubR1 1:1,000; ab70544, Abcam, Cambridge, UK, mouse anti-cdc20 (1:100; sc-5296, Santa Cruz Biotechnology, Santa Cruz, CA, USA), and anti-centromere antibodies (ACA, 1:5,000; kind gift from B. Earnshaw, Welcome Trust Centre for Cell Biology, University of Edinburgh, Edinburgh, UK). Alexa Fluor 488, 568 and 647 (1:1000, Themofisher) were used as secondary antibodies, and DNA was counterstained with 1 µg/ml DAPI (Sigma-Aldrich).

### Quantification of fluorescence intensity at the kinetochores

The fluorescence intensity signal of BubR1 and Cdc20 was measured directly at KTs in a circular ROI (region of interest) using ImageJ and normalized to the intensity signal of ACA in the same ROI. Background fluorescence was measured outside the ROI and subtracted from each KT. The mean values of all quantified KTs quantified were plotted in a scattered dot plot.

### Computational analysis of BubR1 variants

Because there is no experimental structure of the human mitotic checkpoint serine/threonine-protein kinase BubR1 (EC:2.7.11.1) in the Protein Data Bank (http://www.rcsb.org), the molecular structure of the wild-type protein was first constructed by homology modeling using MODELLER software [30] (detailed in Supplementary Methods).

The molecular dynamics simulations were performed to assess the molecular dynamics of the BubR1 proteins in explicit solvent at atomistic resolution. The Amber 18.0 simulation package (parm14SB force field) was used to carry out the optimizations and MD simulations (detailed in Supplementary Methods).

The bioinformatic tools MUpro [31] and I-Mutant 2.0 [32] were used to predict the effect of the currently studied variants (BubR1^R120Q^, BubR1^I147T^, BubR1^R244C^, BubR1^Δ391^ and BubR1^R416Q^) and the control variants (BubR1 ^F175G^, BubR1^F175L^, BubR1^E413K^) on the structural stability of the protein. Starting from the amino acid sequence data, both web servers use a set of machine learning methods to automatically predict protein stability changes upon single-site mutations. These indicators provide fast, quite accurate (77–80% correct previsions) and quantitative previsions of the effects of the variants.

## Results

### Rare *BUB1B* variants are recurrently found in early-onset/familial PrCa and other cancers and carriers of the c.1171_1173del variant share a common ancestor

**Fig. 1:**
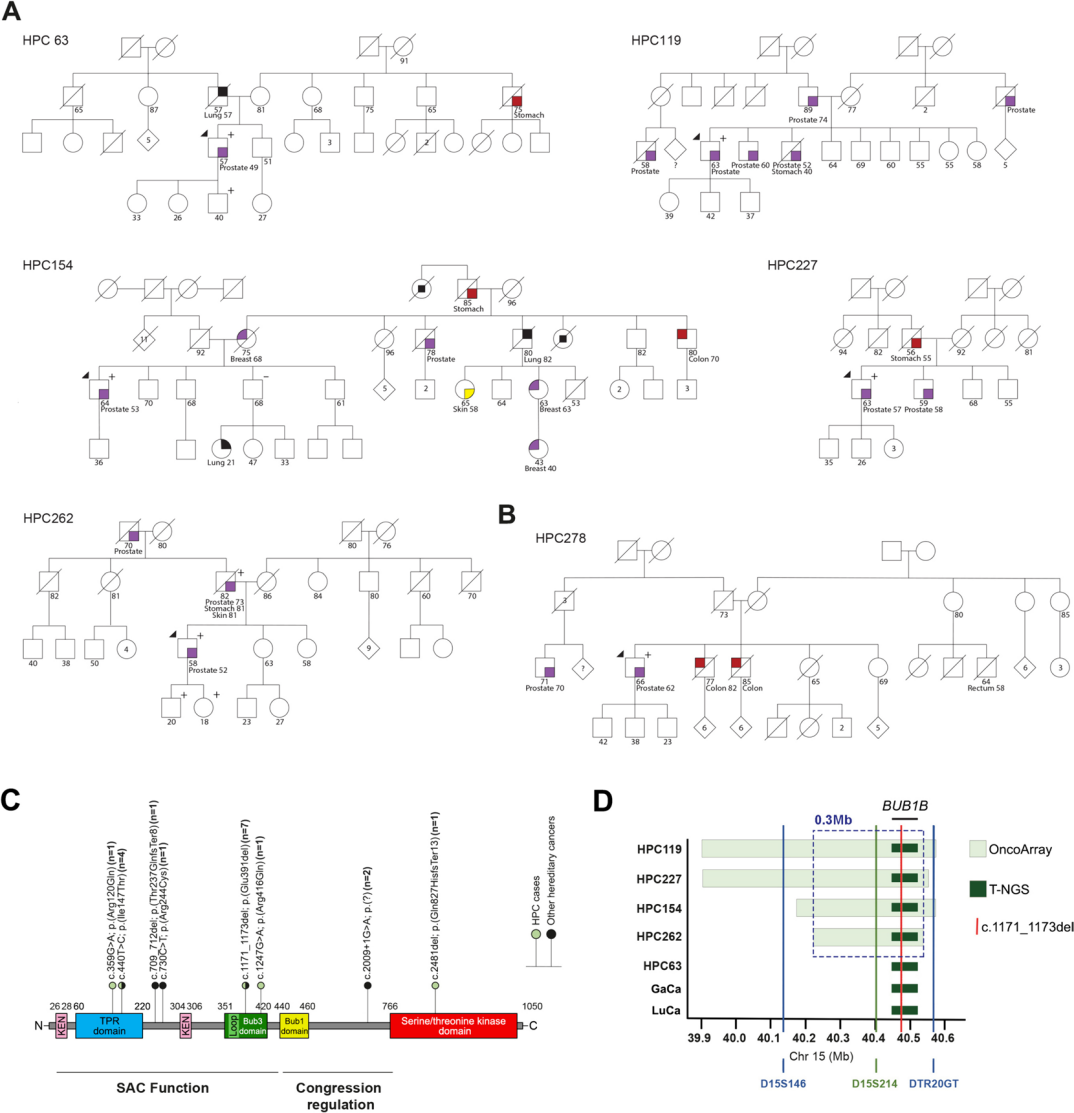
Profile of the genetic variation found in the *BUB1B* gene and flanking genomic region**. A** Pedigree of the patients carrying the *BUB1B* in-frame variant c.1171_1173del; p.(Glu391del). **B** Pedigree of patient HPC278 carrying the *BUB1B* frameshift variant c.2481del, p.(Gln827HisfsTer13). Carrier patients are labeled with a “plus” mark. Squares represent the males, circles the females and diamonds unknown gender. Deceased individuals are represented by a diagonal line through a symbol, and the affected individuals are highlighted by colored symbols. The index case is indicated by an upper left arrow, and the cancer type and age at diagnosis are indicated whenever known. **C** Spectrum and predicted protein consequences of germline *BUB1B* variants identified in HPC patients and patients fulfilling criteria for other hereditary cancers. For each variant, the full number of carrier patients is shown. The main BubR1 functional domains and known functions are highlighted. The lollipop color code indicates the patient group in which variants were identified. **D** Graphical representation of the 0.3 Mb core haplotype shared by the seven carriers of the *BUB1B* c.1171_1173del variant. The dark-green region represents the shared haplotype identified by analysis of T-NGS data, whereas the light-green region represents the haplotypes identified using the high-density SNP Oncoarray genotype data. Microsatellite analysis corroborated the IBD analysis, showing conservation of the microsatellite marker D15S214, and microsatellite variability up- and downstream of the core haplotype boundaries (markers D15S146 and DTR20GT)

The frequency of *BUB1B* variants in our 462 HPC cases was assessed by NGS using a customized targeted gene panel (T-NGS). Overall, six patients were carriers of truncating variants (Table 1, Fig. 1), representing 1.3% of the full HPC series and 1.7% of the patients with early-onset disease (< 56 years at diagnosis). The variant c.1171_1173del, p.(Glu391del), was identified in five patients (Fig. 1A) and the frameshift variant c.2481del, p.(Gln827HisfsTer13), was found in one patient (Fig. 1B). Three of the five families with the recurrent in-frame variant fulfilled both the early-onset and family history criteria (HPC119, HPC154 and HPC262), representing 3.4% of 89 such families. Segregation with the disease was performed in family HPC262, whose index patient had metastatic disease at diagnosis and the carrier father had two additional primary carcinomas (skin and stomach) eight years after the PrCa diagnosis.

**Table 1:** List of *BUB1B* germline variants identified in HPC patients

**Variant position (GRCh37)**	SNP ID	cDNA change	Protein change	MAF^a^	Patient ID	HPC criteria	Other cancers in family^c^ (gender, age^b^)
15:40,462,857	rs1349349252	c.359G > A	p.(Arg120Gln)	0.0016%	HPC450	FH	PrCa (83y)
15:40,468,733	rs763623522	c.440 T > C	p.(Ile147Thr)	0.0083%	HPC369	Age	None
15:40,488,854	rs778590557	c.1171_1173del	p.(Glu391del)	0.0569%	HPC63	Age	GaCa (M, 75y), LuCa (M, 57y)
					HPC119	Age + FH	PrCa (52y), PrCa (60y), PrCa(74y), PrCa (?)
					HPC154	Age + FH	BrCa (F, 68y), BrCa (F, 63y), CoCa (M, 70y), GaCa (M, 86y) LuCa (F, 21y), LuCa (M, 80y), PrCa (78y), SkCa (F, 58y)
					HPC227	FH	GaCa (M, 55y), PrCa (58y)
					HPC262	Age + FH	PrCa (70y), PrCa (73y) + GaCa (81y) + SkCa (81y)
15:40,488,934	rs763272400	c.1247G > A	p.(Arg416Gln)	0.0031%	HPC343	Age	PaCa (M, 62y)
15:40,504,793	rs865779865	c.2481del	p.(Gln827HisfsTer13)	NR	HPC278	FH	CoCa (M, 77y), CoCa (M, 85y), ReCa (M, 58y)

“Age” stands for early-onset diagnosis of PrCa (< 56 years) and “FH” stands for positive family history of *PrCa* BrCa- Breast cancer, *CoCa* Colon cancer, *GaCa* Gastric cancer, *LuCa* Lung cancer, *PaCa* Pancreatic cancer, *ReCa* Rectal cancer, *SkCa* Skin cancer

^a^Minor Allele Frequency (MAF) in male NFE (Non-finish European) obtained from gnomAD; *NR*—Not reported

^b^at diagnosis

^c^1^st^ and 2^nd^ degree relatives

^*^Signet ring cell carcinoma

Of the 875 non-cancer controls genotyped using KASP (Kompetitive Allele-Specific PCR) technology, the variant c.1171_1173del was absent in the 459 male controls, suggesting an increased risk for PrCa development for carriers (OR = 10.98, CI: 0.61–200.39; *p* = 0.074). This ~ ten-fold increased risk for PrCa development is strengthened when compared with the 41 carriers among 36,058 male controls from the Non-Finish European (NFE) population (OR = 9.61, CI: 3.78–24.43; *p* < 0.0001; gnomAD Database – https://gnomad.broadinstitute.org). The frameshift variant c.2481del is not reported in the gnomAD database.

Additionally, three patients were carriers of missense variants (Table 1 and Fig. S1), increasing the frequency of *BUB1B* variants to 1.9% in the whole HPC series and to 2.5% among patients with early-onset disease. Two of these variants [c.359G > A, p. (Arg120Gln); and c.440 T > C, p.(Ile147Thr)] are located in the tetratricopeptide (TPR) domain, while the third variant [c.1247G > A, p.(Arg416Gln)], as well as the recurrent in-frame variant, is located in the Bub3 binding domain (Fig. 1C).

Available data from 1,416 patients screened with the TruSight Cancer Panel at the Department of Laboratory Genetics of IPO Porto for different hereditary cancer syndromes showed nine carriers of *BUB1B* variants (Table 2 and Fig. 1C). The in-frame variant c.1171_1173del, p.(Glu391del), and the missense variant c.440 T > C, p.(Ile147Thr), also identified in HPC patients, were present in two and three patients, respectively, thus being the most frequent *BUB1B* variants identified in this study.

**Table 2:** List of *BUB1B* germline variants identified in non-prostate cancer patients fulfilling criteria for genetic testing

**Variant position (GRCh37)**	SNP ID	cDNA change	Protein change	MAF^a^	Index cancer history (gender, age^b^)	Other cancers in family^c^ (gender, age^b^)
15:40,468,733	rs763623522	c.440 T > C	p.(Ile147Thr)	0.0083%	BrCa (F, 35y)	PrCa (70y)
					BrCa (F, 58y)	BrCa (F, 42y)
					PCC (F, 47y)	None
15:40,476,040	rs992789522	c.709_712del	p.(Thr237GlnfsTer8)	NR	BrCa (F, 32y)	PrCa (55y), OvCa (35y), GaCa (M, 50y)
15:40,476,063	rs867444045	c.730C > T	p.(Arg244Cys)	0.0014%	BrCa (F, 45y)	LuCa (F, 65y)
15:40,488,854	rs778590557	c.1171_1173del	p.(Glu391del)	0.0569%	GaCa* (F, 38y)	CoCa (M, 57y), LuCa (M, 75y)
					LuCa (M, 68y)	LuCa (M, 46y), LuCa (M, 20y)
15:40,498,660	rs1028243319	c.2009 + 1G > A	p.(?)	NR	BrCa (F, 40y, 44y)	PrCa (70y), PrCa (79y), GaCa (M, 47y)
					GaCa* (F, 34y)	None

*BrCa* Breast cancer, *CoCa* Colon cancer, *GaCa* Gastric cancer, *LuCa* Lung cancer, *OvCa* Ovarian cancer, *PCC* Pheochromocytoma

^a^Minor Allele Frequency (MAF) in male NFE (Non-finish European) obtained from gnomAD; NR—Not reported

^b^at diagnosis

^c^1^st^ and 2^nd^ degree relatives

^*^Signet ring cell carcinoma

Among the 875 non-cancer controls genotyped using KASP, the variant c.1171_1173del was found in two females, resulting in an ~ 4.8-fold increased risk for cancer development for carriers (CI: 0.92–24.71; *p* = 0.052).

Considering the high frequency of the c.1171_1173del variant in our cohorts, we investigated a potential founder effect by Identical-by-descent (IBD) analysis. Using data from the T-NGS – custom panel for HPC samples and TruSight Cancer panel for samples from patients with other cancers – a shared haplotype harboring the c.1171_1173del variant was identified among all the seven carriers/families, strongly suggesting a common founder origin (Fig. 1D). To verify whether the shared haplotype extended beyond the *BUB1B* gene, we performed IBD analysis using high-density genome-wide small nucleotide polymorphism (SNP) data available for 374 of the 462 HPC cases as part of the PRACTICAL consortium [26]. IBD analysis identified a shared core haplotype of ~ 0.3 Mb flanking the variant (chr15:40,225,465–40,528,155) in the four carriers profiled, which was not conserved in the 370 non-carriers. Additionally, microsatellite analysis in all carriers and available healthy relatives (Table S3) showed phased haplotypes in two informative families [family HPC262 (Fig. S1) and the GaCa patient family (Fig. S2)], which revealed a conserved region of ~ 113 Kb, comprising the region from the marker D15S214 up to the full *BUB1B* gene (Table S3). This region was also conserved in patients HPC63 and HPC119, and the four remaining carriers (HPC227, HPC154, the LuCa patient, and the healthy son of patient HPC63), despite being heterozygous for the marker D15S2014, harbored the allele consistent with the conserved region. These results corroborate the IBD analysis, showing a conserved microsatellite (D15S2014) within the shared haplotype and microsatellite variability up and downstream of the core haplotype (~ 0.3 Mb) boundaries (D15S146 and DTR20GT) (Fig. 1D), further supporting the existence of a common ancestor.

### Both the recurrent in-frame and missense *BUB1B* variants are predicted to lead to protein instability and impair proper MCC assembly

To determine whether the recurrent in-frame variant and the four missense variants, predicted to result in the proteins p.(Glu391del) (BubR1^Δ391^), (p.Arg120Gln) (BubR1^R120Q^_)_)), p.(Ile147Thr) (BubR1^I147T^), p.(Arg244Cys) (BubR1^R244C^), and p.(Arg416Gln) (BubR1^R416Q^), respectively, may impact the structural stability of the protein, we used the bioinformatic tools Mupro [31] and I-Mutant 2.0 [32]. The predicted free energy changes of two known unstable missense variants, BubR1^E413K^ and BubR1^F175G^ [33–35], and the well-known stable variant BubR1^F175L^ [33, 34], were used as positive and negative cut-off values for interpretation of instability effects. Both bioinformatic tools predict that BubR1^I147T^ greatly destabilizes the protein conformation, while the other missense variants have a rather small effect, if any (Table 3). Mupro also envisages that the in-frame variant BubR1^Δ391^ reduces protein stability, which could then cause protein misfolding, aggregation and degradation.

**Table 3:**
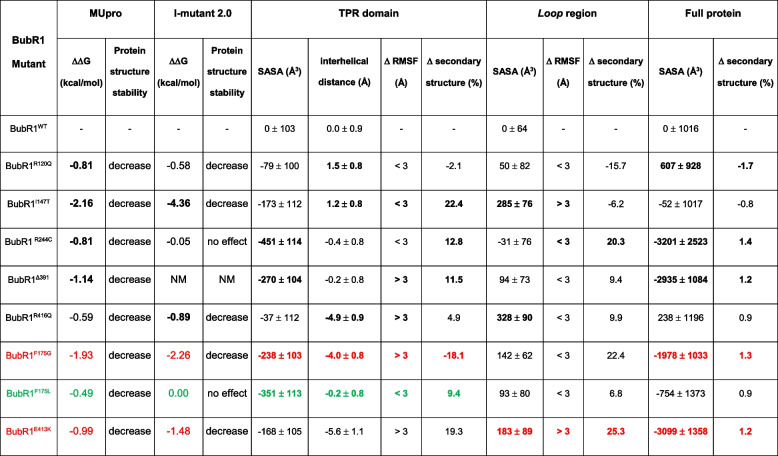
Predicted effect of the different amino acid substitutions on BubR1 stability and structural protein properties

Note: Negative/stable and positive/unstable mutant controls are colored at green and red, respectively, while differences higher than the negative control or statistically valid are highlighted in bold

ΔΔG change in free energy, *TPR* tetratricopeptide, *SASA* solvent accessible surface area, *RMSF* root-mean-square fluctuation, *NM* not measured

**Fig. 2:**
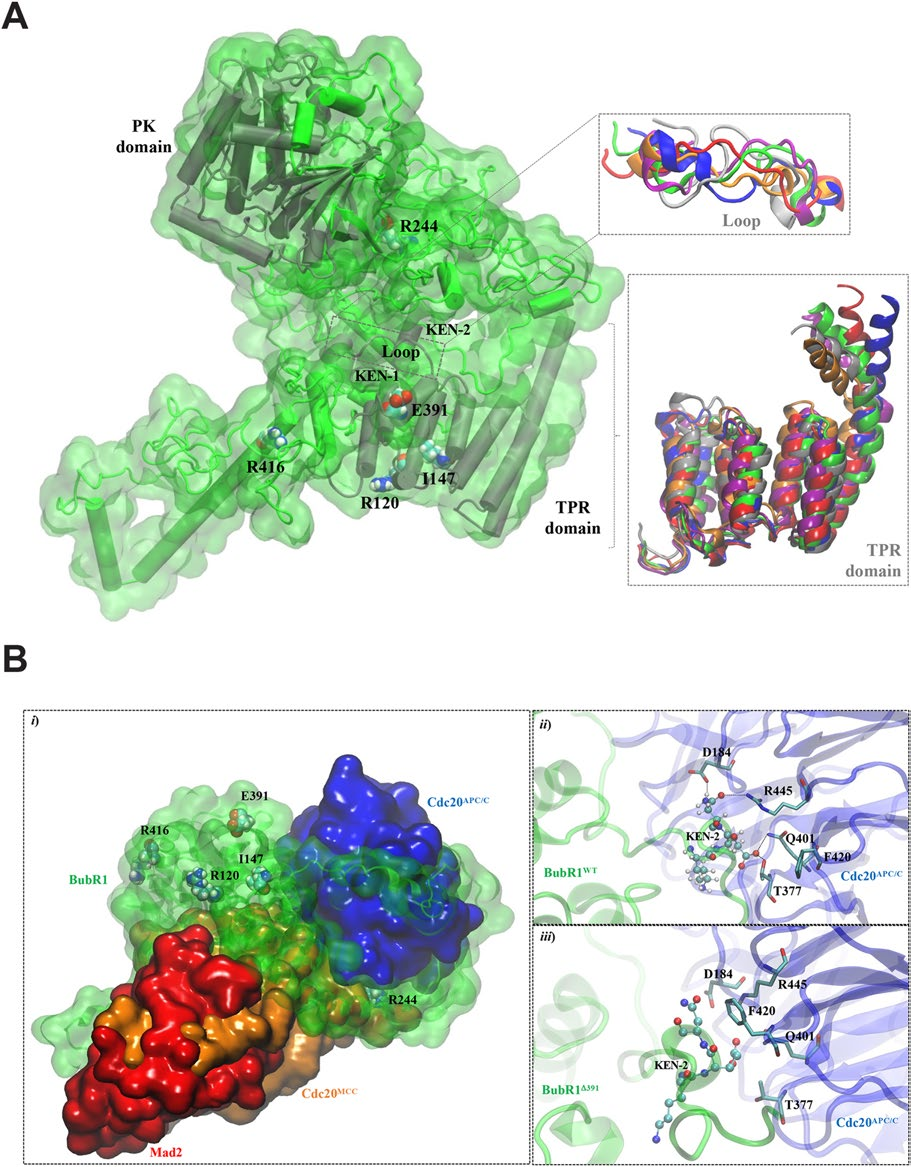
Conformational BubR1 changes and MCC interactions predicted to result from non-truncating *BUB1B* variants.** A** 3D conformational structure of the BubR1^WT^ model showing the localization of the TPR, Loop, KEN boxes, and PK (pseudokinase) domains, as well as the residues predicted to be affected by the missense and in-frame *BUB1B* variants identified in this study. Changes in the conformation of the Loop and TPR domains are highlighted after the superimposition of the BubR1^WT^ (green) model with the BubR1^R120Q^ (red), BubR1^I147T^ (blue), BubR1^R244C^ (silver), BubR1^Δ391^ (purple) and BubR1^R416Q^ (orange) models. **B** 3D structure of the MCC showing the interaction between BubR1^WT^, Mad2, and two Cdc20 molecules, as well as the structural position of the residues predicted to be affected by the missense and in-frame *BUB1B* variants identified in this study (*i*). Close-up of the secondary structure of the KEN-1 motif and surrounding residues showing conformational changes leading to different residue interactions when comparing BubR1^WT^ (*ii*) and BubR1^Δ391^ (*iii*) models

To provide structural details on the impact of the same variants in the BubR1 protein, we performed Molecular Dynamics (MD) simulations on explicit solvated models of BubR1. The structural analysis was focused on specific regions of the highly conserved N-terminus of BubR1, specifically, in two Lys-Glu-Asn motifs (called KEN-1 and KEN-2 boxes), which have been implicated in Cdc20 binding and are required for effective APC/C inhibition and SAC function [36]; in the tetratricopeptide (TPR) domain, which is essential for SAC function via interaction with Knl-1 [34]; and in the Bub3 binding domain (B3BD) [37]. Figure 2A illustrates the molecular model of BubR1^WT^, highlighting the TPR and B3BD regions of the four variants under study by superimposition of the various protein structures. The mutants BubR1^F175G^, BubR1^F175L^ and BubR1^E413K^ were also studied both as structural controls and to validate the computational molecular model. The main structural differences observed in all mutants relative to the BubR1^WT^ are shown in Table 3 (detailed in Tables S4-S7 and Fig. S3). BubR1^Δ391^ and BubR1^R416Q^ show high fluctuation in the atomic position of the TPR residues, while the B3BD region of BubR1^I147T^ has greater mobility than that of BubR1^WT^ (ΔRMSF > 3 Å). Regarding the TPR region, the BubR1^F175G^ is known to disrupt the TPR structure and abolish the binding to Knl-1 (herein used as a positive control) [33, 34]. As the substitution of the same amino acid by a leucine (BubR1^F175L^) maintains the interaction with Knl-1 [33, 34], we used this mutant as a negative control. Our data indicate that the variants BubR1^Δ391^, BubR1^I147T^, BubR1^R244C^ and BubR1^R416Q^ have at least two similar structural changes to the BubR1^F175G^, suggestive of TPR disruption and loss of Knl-1 binding. The BubR1^Δ391^, BubR1^I147T^, and BubR1^R244C^ also show variations in the secondary structure, indicating intrinsic disorder of this region in solution. Additionally, the BubR1^I147T^ loses intramolecular hydrogen bonds between the α-helices of TPR, modifying the overall conformation of TPR, which, subsequently, may also interfere with the interaction with Knl-1 (Fig. S3, detailed in Table S7).

Concerning the B3BD, the structural analysis was focused on the *Loop* (368–379 residues) because this region is a direct APC/C binder and promotes stable association of the BubR1:Bub3 proteins with the APC/C. Indeed, in vitro studies have shown that BubR1^Loop^ mutants have a reduced ability to inhibit the APC/C [35]. Herein, the BubR1^E413K^ located in the B3BD, was used as a positive control due to its known capability to prevent the Bub3 binding and disrupt SAC signaling [35]. Our results denote some similar structural changes in BubR1^I147T^ and BubR1^R416Q^ variants as the BubR1^E413K^, suggesting that these mutants may have comparable effects by interfering with the BubR1:Bub3 association. Despite the proximity of the residue deleted by the in-frame variant (E391) to the Loop, BubR1^Δ391^ does not significantly affect the structure of the Loop region. However, BubR1^Δ391^ shows variations in the solvent exposure area and intramolecular bonds of both KEN-1 and KEN-2 residues (Fig. 2B, Tables S4 and S7). Since KEN-1 is essential for the binding with Cdc20 and Mad2 and the KEN-2 promotes the binding of another Cdc20 [35], these modifications suggest that BubR1^Δ391^ could interfere with the recognition or binding of Cdc20 and Mad2 proteins. All mutants show slight changes in the protein secondary structure, with BubR1^Δ391^, BubR1^R120Q^, and BubR1^R244C^ being the most similar to the positive controls; however, the overall structure is still comparable to the structure of BubR1^WT^.

### In-frame deletions affecting the BubR1 B3BD reduce overall BubR1 abundance and promote proliferation without compromising its recruitment to the kinetochore

Single nucleotide substitutions have been linked to SAC defects caused by severe or partial loss of BubR1 expression in families carrying *BUB1B* biallelic (MVA patients) or monoallelic variants (parents of MVA patients), respectively [19]. This observation led us to hypothesize that the monoallelic *BUB1B* variants found in our study could contribute to (prostate) cancer predisposition through a similar mechanism. To test this hypothesis, and considering their predicted impact on BubR1 structure in silico, we focused on the most frequent variant identified in our study – the in-frame variant c.1171_1173del, p.(Glu391del). For that purpose, we generated prostate cell models with genome editing for the c.1171_1173del variant derived from non-tumorigenic RWPE-1 cells using homology-directed repair (HDR)-mediated CRISPR/Cas9. Since we found no viable cell clones homozygous for the c.1171_1173del variant, we selected monoclonal populations exhibiting a compound heterozygous genotype containing this in-frame *BUB1B* variant (BubR1^Δ391/Δ378−385^) to assess variant’s pathogenicity. Thus, in addition to c.1171_1173del, all the four clones exhibit a second in-frame variant (c.1133_1156del) with a predicted similar impact (located in BubR1 B3BD but outside the *Loop* region; Fig. S4), from which we selected two (named C1 and C2) to use in the following experiments.

**Fig. 3:**
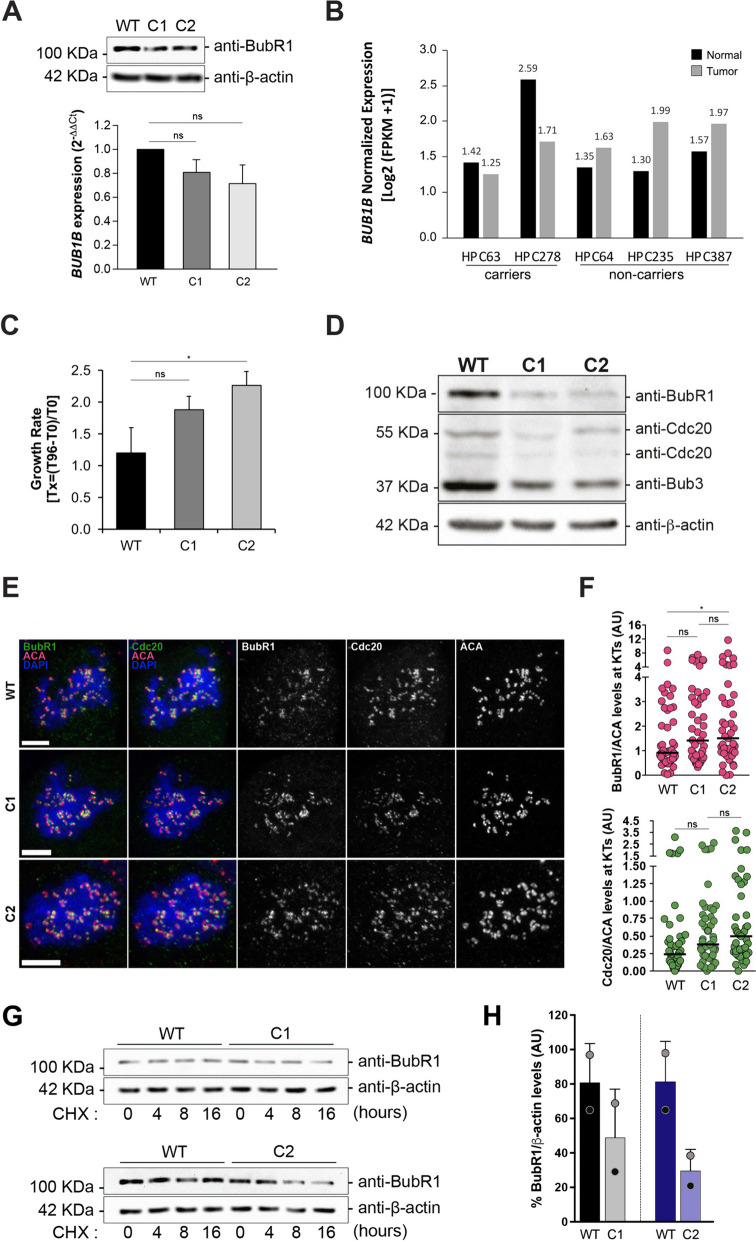
Impact of in-frame deletions in BubR1 B3BD on proliferation and mitosis. **A** Quantification of BubR1 expression at both transcript and protein level in BubR1^WT/WT^ (WT) and BubR1 ^Δ391/Δ378−385^ (C1 and C2) RWPE-1 cells by western-blot and qRT-PCR, respectively. **B** Normalized *BUB1B* expression levels in matched tumor/normal prostate tissues from HPC patients obtained by RNA sequencing. HPC63 and HPC278 are carriers of germline *BUB1B* variants [p.(Glu391del) and p.(Gln827HisfsTer13), respectively] and HPC64, HPC235 and HPC387 are non-carriers of known *BUB1B* variants. **C** Growth rate assessed by the MTT assay at 96 h in culture (*p < 0.05; two tailed, paired* t* test). **D** Protein levels from mitotic cells collected by shake-off upon 16 h of nocodazole treatment. Cell lysates from WT and C1/C2 cell populations were immunoblotted for BubR1 (targeting B3BD), Cdc20 and Bub3. β-actin was used as a loading control. **E–F** Subcellular localization (**E**) and quantification (**F**) of BubR1 and Cdc20 at KT, normalized to ACA levels, upon 4 h of nocodazole treatment. Scale bars, 5 µm. The pool of three independent experiments (45–47 cells) from each cell population is shown (ns- no statistical significance (*p* > 0.05), *p < 0.05; two tailed, unpaired* t* test). **G-H** BubR1 expression levels were assessed by western blot before (0 h) and after blocking de novo protein synthesis with cycloheximide (CHX) for 4 h, 8 h and 16 h (**G**), and quantified by densitometry analysis (**H**). Data were obtained from two independent experiments. A representative blot is shown (**G**), and differences between WT and clonal cell populations are depicted for each WT-paired clonal population after 16h of CHX treatment (**H**). The % of BubR1 expression was obtained by normalizing BubR1 levels to those of the corresponding β-actin and adjusting to the expression levels obtained before CHX treatment (at 0h). For comparison, different colored circles highlight matched clone/WT pairs from the same assay

Quantitative analysis of BubR1 expression both at protein and mRNA levels showed a slight decrease (20–30%) in BubR1 expression in all gene-edited cell clones compared to WT cells (Fig. 3A, Fig. S4). To evaluate whether the established cell models could represent the biology of tumors from carriers of the *BUB1B* in-frame variant c.1171_1173del, we performed RNA sequencing in tumor/normal matched prostate tissues from patient HPC63, carrying the germline variant c.1171_1173del, as well as in prostate tissues from patient HPC278, carrying the frameshift variant c.2481del, and from three additional HPC patients negative for known *BUB1B* germline variants. As observed in our in vitro models, RNA sequencing analysis showed decreased *BUB1B* expression in the prostate cancer tissue relative to the normal counterpart in both *BUB1B* carriers, while tumor/normal matched prostate tissues from non-carriers showed an inverse pattern (Fig. 3B). In vitro, decreased *BUB1B* in C1 and C2 clones led to significantly increased proliferation rates compared with WT cells (Fig. 3C), supporting the oncogenic cancer-predisposing role of this variant.

To gain further insights into the biological mechanism underlying the oncogenic behaviour of *BUB1B*-edited cell clones, we performed western-blot analysis of mitotic protein extracts collected upon cell-cycle arrest with nocodazole for 16 h. In line with decreased mRNA levels, C1 and C2 clones showed a very significant decrease in BubR1 protein expression (Fig. 3D) and a moderate and a slight decrease in Cdc20 (two isoforms) and Bub3 expression levels, respectively, suggestive of premature disassembly and degradation of the MCC components. As free MCC levels (not KT-bound) are critical for SAC signaling [13], we investigated whether the “instability” of the MCC was due to improper recruitment of BubR1 and Cdc20 to the KTs. For this purpose, we quantified the expression levels of BubR1 and Cdc20 at KTs in the absence of microtubules (unattached KTs) in randomly selected mitotic cells treated with nocodazole for 4 h. Similar expression levels were observed in edited and control cells (Figs. 3E-F), although edited cells showed a tendency for higher expression of both BubR1 and Cdc20 at KTs, reaching statistical significance in the C2 population.

To assess whether reduced BubR1 protein levels in *BUB1B* gene-edited cell populations may be caused by increased protein degradation, as predicted in silico, we measured the half-life of BubR1 by cycloheximide (CHX) chase in both WT and gene-edited cell clones. We consistently observed lower BubR1 levels in both C1 and C2 cell populations in every time point comparing with WT cells (Fig. 3G and Fig. S5), which are more pronounced after 16 h of CHX treatment (Fig. 3H). This tendency is observed within each assay, despite the variability observed when combining the two experiments. The same tendency was observed in a 3^rd^ independent clone with the same genotype (C3; Fig. S5).

These results suggest that, as predicted in silico, the recurrent *BUB1B* in-frame deletion c.1171_1173del (BubR1^Δ391^) compromises the stability of the MCC but not the ability to recruit BubR1-Cdc20 to unattached KTs.

### In-frame deletions affecting the BubR1 B3BD impair SAC signaling and trigger chromosomal instability, driving resistance to Taxol treatment

**Fig. 4:**
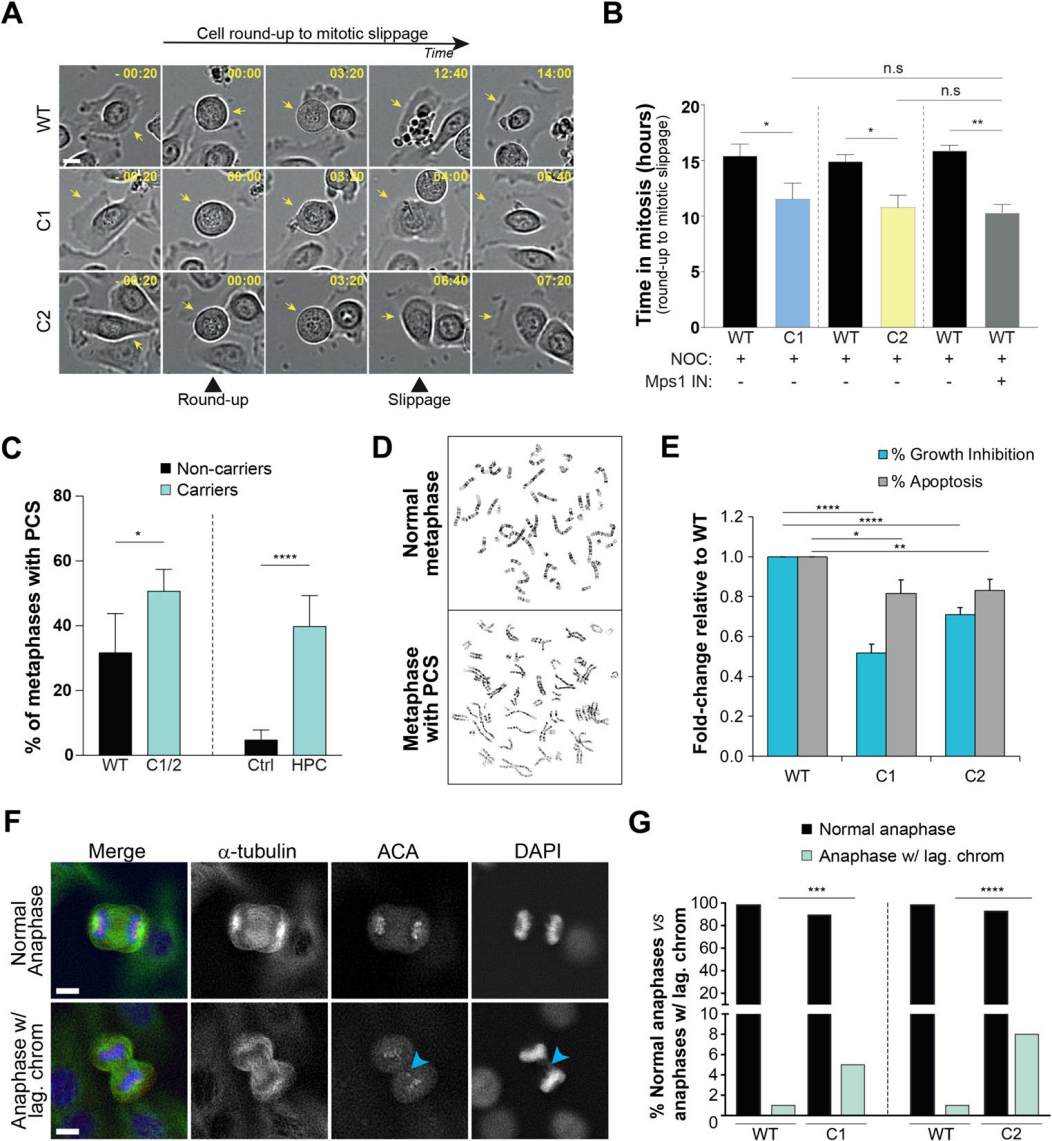
*BUB1B* in-frame variants in B3BD impair SAC robustness leading to PCS and Taxol-resistant chromosomal instability**. A** Representative images of phase contrast microscopy time-series illustrating mitotic entry (cell round-up), mitotic arrest by nocodazole treatment and mitotic exit in WT and *BUB1B*-edited cells (C1 and C2). Images were acquired every 20 min. Scale bar, 10 µm. Time, hr:min. **B** Quantification of mitotic duration of the cells represented in A. Bars indicate mean values and the error bars represent the SD from three independent experiments (*p < 0.05, **p < 0.01, two tailed, unpaired* t* test). **C** Bar plot of the frequency of metaphase cells with PCS comparing *BUB1B*-edited and WT cells (*left*) and human blood metaphase cells in age-matched male controls compared to HPC patients carrying the *BUB1B* c.1171_1173del variant (*right*). Error bars represent the SD of the mean of three independent experiments (*p < 0.05, ****p < 0.0001, two tailed, unpaired* t* test). At least 100 metaphase cells were analyzed per condition.** D** Representative images of normal metaphase and PCS events in carriers of the *BUB1B* c.1171_1173del variant. **E** Impact on growth inhibition and apoptosis after 96 h of treatment with Taxol (10 nM; *p < 0.05, **p < 0.01, ****p < 0.0005, two tailed, paired* t*-test). **F** Representative images of anaphase cells immunostained with antibodies against α-tubulin and centromere proteins (ACA), depicting normal anaphase and chromosome missegregation in anaphase (lagging chromosome) highlighted by a blue arrow. DNA was counterstained with DAPI. Scale bars, 10 µm. **G** Quantification of the percentage of normal anaphases *vs* anaphases with lagging chromosomes from a pool of six independent experiments (***p < 0.001, ****p < 0.0001, Chi-squared test)

To test SAC function in our cell models, we performed a well-established assay based on the measurement of mitotic duration in the presence of persistently unattached KTs upon treatment with nocodazole. Our live-cell analysis revealed that C1 and C2 cells exit mitosis significantly earlier (11.6 ± 1.4 h and 10.8 ± 1.1 h, respectively) than WT cells (15.4 ± 1.1 h and 14.9 ± 0.7 h, respectively) (Fig. 4A-B). Moreover, the impact of the *BUB1B* variants on SAC function was as severe as the acute SAC disruption obtained upon Mps1 inhibition (Fig. 4B). Cytogenetic examination and quantification of premature chromatid separation (PCS) showed a significant increase in PCS in both *BUB1B*-edited clones (38–56%) compared to WT cells (32%) (Fig. 4C-D). Similarly, HPC patients carrying the monoallelic *BUB1B* c.1171_1173del variant exhibited 34–54% PCS metaphases compared to 0.75–8.74% found in age-matched male controls (Table S8, Fig. 4C). High percentages of PCS were also observed in patient HPC278, carrying the frameshift variant c.2481del, in patient HPC450, carrying the missense variant c.359G > A, and in the healthy sons of patients HPC63 and HPC262, both carrying the c.1171_1173del variant (Table S8).

The efficacy of taxane-based chemotherapies, currently approved by the United States Food and Drug Administration (FDA) for the treatment of several advanced cancers, including metastatic castration-resistant prostate cancer [38], is limited by intrinsic and acquired drug resistance associated with higher CIN [20, 21]. Therefore, we questioned whether increased PCS in *BUB1B* carriers would be associated with increased CIN and whether CIN levels would correlate with response to Taxol treatment. We observed a dramatic resistance to Taxol-induced growth inhibition and apoptosis in *BUB1B*-edited cells compared with WT cells (Fig. 4E). Complementarily, immunofluorescence analysis revealed a significantly higher percentage of lagging chromosomes in anaphase in *BUB1B*-edited cells (Fig. 4F-G). Altogether, these observations establish a link between BubR1 in-frame deletions affecting B3BD, SAC defects, CIN and Taxol resistance, sustaining the pathogenicity of the recurrent variant c.1171_1173del (BubR1^Δ391^).

## Discussion

In this work, we identified nine carriers of rare germline variants in the *BUB1B* gene among 462 PrCa patients fulfilling the criteria for hereditary prostate cancer. This makes *BUB1B* one of the most frequently mutated genes described in our HPC patients, accounting for 1.9% of the early-onset/familial PrCa patients and 2.5% of the patients with early-onset disease (diagnosis < 56 years). Additionally, 0.6% of the cancer patients fulfilling the criteria for genetic testing for other hereditary cancer syndromes were also carriers of rare *BUB1B* germline variants, following the *pan*-cancer profile observed for known moderate- to high-penetrance risk genes.

Considering the broad dissemination of NGS technology throughout genetic laboratories worldwide, the American College of Medical Genetics and Genomics and the Association for Molecular Pathology (ACMG/AMP) have published guidelines for variant interpretation in genes associated with Mendelian disorders, narrowing the translatability of genetic findings [39]. However, in genes associated with recessive syndromes (caused by two deleterious variants), as is the case of *BUB1B* in MVA, the pathogenicity of monoallelic variants may be harder to clarify, since the effect of isolated variants may not reflect that of variant combination. *BUB1B*, however, has an advantage over other genes in this category since heterozygous *BUB1B* variants are associated with an autosomal dominant trait – PCS (OMIM # 176,430). In fact, fundamental studies have defined a link between loss of BubR1 expression, mitotic checkpoint defects, PCS and CIN [17, 18]. Additionally, both truncating and non-truncating variants in *BUB1B* have been associated with loss of protein expression and mitotic defects, determinants of the MVA phenotype [19, 40, 41]. Nevertheless, despite the potential of spindle checkpoint genes as tumor suppressors and the first validation of a causal link between *BUB1B* variants and lung and colorectal cancer development in BubR1^±^ mouse models in 2004 [41], the cancer-predisposing role of *BUB1B* remains largely elusive.

Several studies have explored the functional relevance of BubR1 variants identified in MVA patients, which mainly cluster in the kinase domain (C-terminal) [19, 42]. In our study, aside from the frameshift variant c.2481del and the splicing variant c.2009 + 1G > A, the *BUB1B* variants identified, both in HPC patients and in patients with other cancers, cluster at or near the TPR and B3BD domains (N-terminal). Thus, we have started by investigating the potential consequences of the identified *BUB1B* variants at the protein level using in silico molecular modeling and molecular dynamics simulations of all non-truncating *BUB1B* variants, using known BubR1 mutants as controls. Our analysis shows that all the identified variants are predicted to lead to mild to severe loss of BubR1 stability and to TPR disruption, probably impairing Knl-1 binding and leading to SAC dysfunction. To validate this assumption, we focused on the most frequent variant identified in our study, and one of the two variants here described, and ever reported, to be recurrently found in cancer patients – c.1171_1173del (BubR1^∆391^) – representing 38.9% of all *BUB1B* variant carriers and 55.5% of HPC index patients who carry a *BUB1B* variant. Hahn et al. (2016), in an attempt to clarify the phenotypic implications of two *BUB1B* variants (c.1171_1173del and c.2834G > A) identified in the germline of early-onset colorectal cancer cases, have induced overexpression of both mutants and WT *BUB1B* in HEK293 and HeLa cells and found no differences in protein expression, localization (at KTs), or Bub3 binding between the three cell models [8]. Aiming to look beyond Hahn et al. observations, we used CRISPR/Cas9 to generate clonal cell populations derived from the non-tumorigenic RWPE-1 prostate cells carrying the variant c.1171_1173del (BubR1^Δ391^). Despite the high success rate of genomic editing by homology-directed repair (~ 26.8%; detailed in Supplementary Methods), heterozygous (BubR1^WT/Δ391^) or homozygous (BubR1^Δ391/Δ391^) clonal populations were not found. Instead, the four clonal populations that persisted to grow harbored a compound heterozygous genotype (BubR1^Δ391/Δ378−385^) in which the second variant was also in-frame and localized in B3BD (c.1133_1156del). Although speculative, it is possible that, as described for other *BUB1B* truncating variants, the homozygosity for the c.1171_1173del, or its co-occurrence with a truncating variant, are not compatible with life, in which case a pathogenic role would be attributed to the c.1171_1173del. On the other hand, the absence of heterozygous cell populations (BubR1^WT/Δ391^) may indicate a technical issue related with the CRISPR/Cas9 approach used. It is possible that finetuning the Cas9 activity or, alternatively, using a high fidelity Cas9 would improve monoallelic gene-editing [43, 44]. However, considering the recessive nature of *BUB1B* variants and the fine regulation of BUBR1 activity, with a barely measurable phenotypic impact observed in the studied compound heterozygous cell populations (BubR1^Δ391/Δ378−385^), it is also possible that monoallelic gene-editing would result in unperceived phenotypic changes in the timeframe of these assays.

The established cell models validate the observations from Hahn et al. [8] by showing that in cells carrying the variant c.1171_1173del, both BubR1 and Cdc20 are efficiently recruited to unattached KTs, yet we further show that this is not sufficient to ensure proper SAC function and chromosome segregation fidelity, probably as a result of a decrease in both protein expression and stability of KT-independent MCC. As a consequence, *BUB1B*-edited cells showed an increase in both PCS and lagging chromosomes, markers of CIN [12, 17], which correlated with decreased sensitivity to Taxol-induced growth inhibition, as observed in other cancer models [20, 45, 46]. Although we cannot exclude possible cumulative effects of the two in-frame variants present in our in vitro cell models, in vitro PCS levels are consistent with increased PCS levels observed in lymphocytes derived from *BUB1B* carriers and with the autosomal dominant effect of heterozygous *BUB1B* variants described for the PCS trait, strongly suggesting that the variant c.1171_1173del is sufficient to trigger oncogenic transformation. Additionally, *BUB1B*-edited clonal cell populations showed a partial to severe loss of *BUB1B* transcripts, recapitulating the observations from RNAseq analysis of tumor samples from patients carrying *BUB1B* variants, and also a severe loss of protein abundance, respectively, leading to proliferation-prone premature mitotic exit in a checkpoint-dependent manner, similar to what has been described for MVA variants [19] and supporting the hypomorphic nature of the variant c.1171_1173del. Although increased *BUB1B* expression has been described as a common mechanism underlying carcinogenesis in different sporadic cancer types [47–49], our study shows that a different oncogenic-driver underlying mechanism operates in cells with decreased *BUB1B* expression triggered by a rare monoallelic variant. Moreover, our observation of decreased Taxol sensitivity in *BUB1B*-edited cells is in line with the finetuned balance between CIN levels and cell fate, where high CIN levels compromise response to Taxol-based therapies [45, 46], potentially highlighting the ineffectiveness of Taxol-based therapies for the treatment of carcinomas arising in carriers of “deleterious” *BUB1B* variants.

Notwithstanding the identification of a core haplotype of 0.3 Mb among carriers of the variant c.1171_1173del, sustaining the hypothesis of a founder effect in northern Portugal, it is possible that the high fluctuation in the percentage of PCS among carriers (19–70%) is related to genetic variation outside the boundaries of the shared haplotype, as reported for different haplotypes found in Japanese families with PCS syndrome [18]. Further studies assessing the prevalence of carriers and the corresponding haplotypes across diverse genetic backgrounds holds immense promise in elucidating the origins of this variant and expanding our comprehension of its pivotal role in driving cancer development.

## Conclusions

Our data provide solid evidence that monoallelic *BUB1B* variants are sufficient to cause a large spectrum of subclinical progressive chromosomal instability that predisposes carriers to (prostate) cancer development, supporting the classification of the variants identified in this study as “likely pathogenic” and pinpointing *BUB1B* as a (*pan*)cancer predisposing gene. Altogether, our studies suggest that patients carrying the variant c.1117_1173del, or potentially other “deleterious” variants in the *BUB1B* gene, may benefit from pretherapeutic assessment of CIN/PCS to optimize treatment stratification and clinical trial design.

### Supplementary Information

Additional file 1. Supplementary Methods.

Additional file 2. Supplementary Tables (Tables S1-S8).

Additional file 3. Supplementary Figures (Figures S1-S5).

## Data Availability

All data and associated protocols are included in the Manuscript and Supplementary Information and available to the readers. Cell lines generated in this study are available upon request.

## Abbreviations

ACMG/AMP: American College of Medical Genetics and Genomics and the Association for Molecular Pathology
B3BD: Bub3 binding domain
BubR1: Budding uninhibited by benzimidazole-related 1
CIN: Chromosomal instability
HPC: Early-onset/familial (hereditary) PrCa
KT: Kinetochore
MVA: Mosaic Variegated Aneuploidy
MCC: Mitotic checkpoint complex
PCS: Premature Chromatid Separation
SAC: Spindle Assembly Checkpoint
TPR: Tetratricopeptide (domain)
