# Supplementary material for: BUB1B monoallelic germline variants contribute to prostate cancer predisposition by triggering chromosomal instability
Source: J Biomed Sci. 2024 Jul 16;31:74. doi: 10.1186/s12929-024-01056-z (PMC11251299; doi:10.1186/s12929-024-01056-z)
Supplement: Supplementary file 1 — Additional file 1. Supplementary Methods. [file 12929_2024_1056_MOESM1_ESM.docx]

# Supplementary Methods

# *BUB1B* monoallelic germline variants contribute to prostate cancer predisposition by triggering chromosomal instability

Maria P. Silva^1,#^, Luísa T. Ferreira^1,#^, Natércia F. Brás^2^, Lurdes Torres^1,3^, Andreia Brandão^1^, Manuela Pinheiro^1^, Marta Cardoso^1^, Adriana Resende^1,3^, Joana Vieira^1,3^, Carlos Palmeira^4^, Gabriela Martins^4^, Miguel Silva^1,3^, Carla Pinto^1,3^, Ana Peixoto^1,3^, João Silva^1,3^, Rui Henrique^5^, Sofia Maia^1^, Helder Maiato^6,7,8^, Manuel R. Teixeira^1,3,9,&^, Paula Paulo^1,&;^*

^1^Cancer Genetics Group, IPO Porto Research Center (CI-IPOP) / RISE@CI-IPOP (Health Research Network), Portuguese Oncology Institute of Porto (IPO Porto) / Porto Comprehensive Cancer Center, Porto, Portugal;

^2^LAQV, REQUIMTE, Department of Chemistry and Biochemistry, Faculty of Sciences, University of Porto, Porto, Portugal;

^3^Department of Laboratory Genetics, Portuguese Oncology Institute of Porto (IPO Porto) / Porto Comprehensive Cancer Center, Porto, Portugal;

^4^Department of Immunology, Portuguese Oncology Institute of Porto (IPO Porto) / Porto Comprehensive Cancer Center, Porto, Portugal;

^5^Department of Pathology, Portuguese Oncology Institute of Porto (IPO Porto) / Porto Comprehensive Cancer Center, Porto, Portugal;

^6^Chromosome Instability & Dynamics Group, Instituto de Investigação e Inovação em Saúde, University of Porto / Porto Comprehensive Cancer Center, i3S, Porto, Portugal;

^7^Cell Division Group, Experimental Biology Unit, Department of Biomedicine, Faculty of Medicine, University of Porto, Porto, Portugal;

^8^Instituto de Biologia Molecular e Celular, University of Porto, Porto, Portugal;

^9^School of Medicine and Biomedical Sciences (ICBAS), University of Porto, Porto, Portugal.

^#,&^ Equal contributions

*Corresponding author, Paula Paulo

**Email:**  paula.paulo@ipoporto.min-saude.pt

**Targeted next-generation sequencing (T-NGS)**

Variants with a frequency above 10% in the whole dataset, a minor allele frequency (MAF) >0.1% in the non-Finnish European population (NFE; gnomAD database [1]; https://gnomad.broadinstitute.org) or in the Iberian population (IBS, 1000G phase III project database [2]) or predicted to be synonymous were excluded. Additionally, non-synonymous variants predicted to be deleterious by less than 9 of the 11 pathogenicity predictors (SIFT, Polyphen2-HDIV, LRT, MutationTaster2, MutationAssessor, FATHMM, MetaSVM, MetaLR, CADD, VEST4, and MutPred) or a conservation score less than 0.5 in two of the four conservation tools (PhyloP, phastCons, GERP++, and SiPhy), all available at dbNSFP (http://database.liulab.science/dbNSFP#database [3]), were excluded. All the described *BUB1B* variants were validated by Sanger sequencing.

**Analysis of RNA sequencing data from FFPE tumor samples**

The analysis of *BUB1B* expression was performed according to the protocol described by Pertea et al. (2016) [4]. Briefly, the raw sequence reads were aligned to the human reference genome (GRCh38) using HISAT2 v2.2.1 [5]. The HISAT2 index for the reference genome was built using the same reference genome. The resulting SAM files were converted to sorted and indexed BAM files using Samtools v1.11 [6]. The alignments were assembled into full and partial transcripts and quantified using StringTie v2.1.4 [4]. The reference annotation file (Homo_sapiens.GRCh38.108.gtf) obtained from Ensembl was used as a guide for transcript assembly. The expression levels of each gene and transcript were quantified as FPKM (fragments per kilobase of transcript per million mapped reads).

**Generation of prostate *in vitro* cell models carrying the BubR1^Δ391^ variant**

The design of gRNAs targeting *BUB1B* exon nine, and the HDR donor template was performed using the Alt-R HDR Design Tool from IDT (https://www.idtdna.com/pages/tools/alt-r-crispr-hdr-design-tool). The HDR donor included the *BUB1B* in-frame deletion and homologous arms with 40 bp in each side of the variant. A synonymous variant (G>A) was also introduced to modify the PAM and prevent Cas9 recutting of the target after gene editing. The sequences of the gRNAs and HDR donor template used are listed in **Table S8**.

For paired nicking experiments, each RNP-bound gRNA was formed separately, and two RNPs were mixed at an equal molar ratio prior to adding to the cells at the time of transfection. For cationic lipid delivery of RNP complexes, we used the CRISPRMAX reagent (Thermo Fisher Scientific), and the reverse transfection method was used for delivery to RWPE-1 cells in a 6-well plate. To increase the activity of the homology-directed repair (HDR) system, after 48 h, cells were collected, and 5x10^5^ cells were seeded in a 6-well plate for cell cycle synchronization for 16 h with nocodazole (0.033 µM, Sigma-Aldrich, Burlington, MA, USA), followed by 3 h with aphidicolin (2 µg/mL, Sigma-Aldrich) [7,8]. The efficiency of enrichment for cells in S+G2/M was evaluated by flow cytometry with propidium iodide staining, as described by others [9]. Synchronized cells were transfected with 7.5 µg (180 pmol) of Alt-R HDR template donor (from IDT) containing the *BUB1B* c.1171_1173del variant with Xfect transfection reagent (Clontech, Montain View, CA, USA) according to the manufacturer’s instructions. An Alt-R HDR enhancer (3 mM, from IDT) was co-delivered with the Alt-R HDR template to the cells to a final concentration of 30 µM [10]. The 6-well plate was transferred to a 32°C, 5% CO_2_ incubator (cold-shock) for 4 h [11], after which both nanoparticle complexes and HDR enhancer were removed from cells and replaced by KSF complete medium containing 30 µM HDR enhancer only. The plates were returned to the 32ºC, 5% CO_2_ incubator for another 16 h, after which the medium was replaced by complete KSF medium for optimal cell response. The cells were kept at 32ºC for another 24 h and then maintained at 37ºC. Monoclonal populations were isolated by the serial dilution technique in 384-well plates. After expansion in 96-well plates, replicates from clonal populations were genotyped using Sanger sequencing and screened for HDR-mediated gene-specific editing using Synthego’s ICE (Inference of CRISPR Edits) tool. Of the 153 isolated clones, ~26.8% showed genomic integration of the synonymous variant included in the HDR donor, and approximately half of those (19/41) had 30-50% of the sequencing reads with insertion of the c.1171_1173del (predicted by ICE). Among those, the large majority (13/19) entered a senescence/apoptotic state and were discarded. Of the six remaining clones that were viable and expanded, two were discarded for having a complex genotype, eventually as a result of a polyclonal population, and all four remaining clones harbored a second in-frame variant (c.1133_1156del) predicted to also affect the B3BD [p.(Pro378_His385del)] (**Figure S4**). Two of these four clones were selected for further studies, namely, clone #47 (referred to as C1) and clone #131 (referred to as C2). Quantitative analysis of the *BUB1B* transcriptomic profile by droplet digital PCR showed that wild-type (WT) *BUB1B* was not expressed, 54-58% of the *BUB1B* transcripts harbored the c.1171_1173del variant and 42-46% harbored the c.1133_1156del variant.

**Droplet digital PCR (ddPCR)**

Cells at ~90% confluence in T25 flasks were nocodazole-arrested (0.5 µM, Sigma-Aldrich) for 16 h, washed using D-PBS (GRiSP, Porto, Portugal) and trypsinized with TrypLE™ Express Enzyme (Gibco, Thermo Fisher Scientific). Cells were harvested and centrifuged at 300×g for 5 min and the pellet was washed twice using D-PBS before RNA extraction using the RNeasy® Mini Kit (Qiagen, Frederick, MD, USA) following the manufacturer’s instructions. RNA was quantified using a Qubit RNA Assay (Thermo Fisher Scientific, Carlsbad, CA, USA). For cDNA synthesis, 1 µg of RNA was subjected to reverse transcription with random hexamers using the H-minus RevertAid cDNA synthesis kit (Thermo Fisher Scientific) according to the manufacturer’s instructions. ddPCR was performed with the QuantStudio 3D Digital PCR System (Applied Biosystems, Waltham, MA, USA), and assays were run in duplicate for each sample. The reaction mix for two chips was composed of 17.4 μl of QuantStudio 3D Digital PCR Master Mix v2, 1.74 μl of the respective primer/probe assay, 3.66 μl of DNase/RNase-free water and 12.0 μl of cDNA (~1 ng/μl). Then, 14.5 μl of the reaction mixture was loaded on a QuantStudio 3D Digital PCR Chip v2 using QuantStudio 3D Digital PCR Chip Loader. The chips were placed in a GeneAmp PCR System 9700 for thermal cycling under the following conditions: 96°C for 10 min, followed by 39 cycles of a two-step thermal profile at 60°C for 2 min, and 98°C for 30 s and a final extension step at 60°C for 2 min. Thereafter, the chips were read with the QuantStudio 3D Digital PCR Instrument and analyzed using the QuantStudio 3D AnalysisSuite Cloud Software (Thermo Fisher Scientific; https://apps.thermofisher.com/quantstudio3d/).

**Computational analysis of BubR1 variants**

***Molecular modeling*.** The BubR1 (chain S) present in the APC/C-MCC complex (PDBID 5LCW), the BubR1 (PDBID 2 WVI) and the HMP-1 M domain (PDBID 5H5M), the Karyopherin nuclear state - RNA binding protein (PDBID 3ICQ) and the Bub1 kinase domain (PDBID 4QPM) were used as templates to generate the model (with discrete optimized protein energy (DOPE) varying between -0.59 to 0.80). On the other hand, the 3D structure of BubR1 predicted by the AlphaFold v2.0 (AF-O60566-model), an artificial intelligence approach used across diverse applications equally well compared to experimentally determined structures [12], was also used in further studies. Both models have high yield quality factors of ERRAT analysis (http://servicesn.mbi.ucla.edu/ERRAT/) (88.4 and 94.3, respectively), and their reliability has also been evaluated by their superimposition with the N-terminus of BubR1 present in the APC/C-MCC complex (PDBID 6TLJ). Although the two fit rather well in the crystallographic complex, the latter maximizes the interactions with the Cdc20^MCC^, Cdc20^APC/C^ and Mad2 proteins and represents an average of protein conformational substates, so it was used in further simulations. Starting from the wild-type (BubR1^WT^) geometry, various BubR1 mutants (R120Q, F175G, F175L, I147T, R244C, Δ391, E413K and R416Q) were modelled using the X-leap tool of the Amber 18.0 simulation package [13].

The interaction ability between the SAC components and the APC/C co-activator Cdc20 was also assessed by modeling a tetrameric complex composed of BubR1^1-420^, Cdc20^MCC^, Cdc20^APC/C^ and Mad2. The X-ray APC/C-MCC complex (PDBID 6TLJ) was used as the starting geometry because it has the two well-defined Cdc20 subunits, the Mad2 and the N-terminus of BubR1 [14]. The latter was modeled until the amino acid 420 due to its reported essential role in SAC function [15].

***Molecular Dynamics (MD) simulations.*** Several MD simulations at 310 K were performed to assess the molecular dynamics of the BubR1 proteins in explicit solvent at atomistic resolution. The Amber 18.0 simulation package (parm14SB force field) was used to carry out the optimizations and MD simulations. The force field parameters developed by Sticht et al*.* (2006) [16] and Kaxiras et al. (2012) [17] were assigned for the modified phosphorylated amino acids (phosphoserine and phosphothreonine) and acetylated lysine, respectively. The protonation states of the ionizable residues were determined by the PropKa tool [18]. Re-equilibrated TIP3P water molecules were used, filling a rectangular box with a minimum distance of 15 Å between the box faces and any atom of each system. First, each system was minimized in two steps: a harmonic restraint of 20 kcal mol^-1^ Å^-2^ was kept on protein for 10000 steps, and then, an unrestrained minimization for 30000 steps was performed with the steepest descent and conjugate gradient methods. Then, they were equilibrated for 100 ps, followed by 100 ns of production MD simulation. To increase the sampling, 3 replicas, starting from different initial velocities, were simulated for each system. The pressure and the temperature were controlled by using the Berendsen barostat and the Langevin thermostat [19]. The SHAKE algorithm [20] was employed to constrain the bond lengths involving hydrogen atoms. Periodic boundary conditions were considered. Non-bonded interaction pairs were calculated within 10 Å. Beyond that, Coulomb interactions were analyzed by the Particle-Mesh Ewald (PME) algorithm [21], and vdW interactions were truncated. The MD trajectories were analyzed with the CPPTRAJ module [22] of the Amber 18.0 simulations package, combined with the visual molecular dynamics (VMD 1.9.2) program for visualization, analysis and image rendering [23].

**References**

1. Karczewski KJ, Francioli LC, Tiao G, Cummings BB, Alföldi J, Wang Q, et al. The mutational constraint spectrum quantified from variation in 141,456 humans. *Nature*. 2020;**581**:434–443. doi: 10.1038/s41586-020-2308-7.

2. Fairley S, Lowy-Gallego E, Perry E, Flicek P. The International Genome Sample Resource (IGSR) collection of open human genomic variation resources. *Nucleic Acids Res*. 2020;**48**(D1):D941–D947. doi: 10.1093/nar/gkz836.

3. Liu X, Li C, Mou C, Dong Y, Tu Y. dbNSFP v4: a comprehensive database of transcript-specific functional predictions and annotations for human nonsynonymous and splice-site SNVs. *Genome Med*. 2020;**12**:103. doi: 10.1186/s13073-020-00803-9.

4. Pertea M, Kim D, Pertea GM, Leek JT, Salzberg SL. Transcript-level expression analysis of RNA-seq experiments with HISAT, StringTie and Ballgown. *Nat Protoc*. 2016;**11**:1650–1667. doi: 10.1038/nprot.2016.095.

5. Kim D, Paggi JM, Park C, Bennett C, Salzberg SL. Graph-based genome alignment and genotyping with HISAT2 and HISAT-genotype. *Nat Biotechnol*. 2019;**37**:907–915. doi: 10.1038/s41587-019-0201-4.

6. Danecek P, Bonfield JK, Liddle J, Marshall J, Ohan V, Pollard MO, et al. Twelve years of SAMtools and BCFtools. Gigascience. 2021;**10**:giab008. doi: 10.1093/gigascience/giab008.

7. Heyer WD, Ehmsen KT, Liu J. Regulation of homologous recombination in eukaryotes. *Annu Rev Genet*. 2010;**44**:113–139. doi: 10.1146/annurev-genet-051710-150955.

8. Lin S, Staahl BT, Alla RK, Doudna JA. Enhanced homology-directed human genome engineering by controlled timing of CRISPR/Cas9 delivery. *Elife*. 2014;**3**:e04766. doi: 10.7554/eLife.04766.

9. Vasconcelos-Nóbrega C, Pinto-Leite R, Arantes-Rodrigues R, Ferreira R, Brochado P, Cardoso ML, et al. In vivo and in vitro effects of RAD001 on bladder cancer. *Urol Oncol*. 2013;**31**:1212–21. doi: 10.1016/j.urolonc.2011.11.002.

10. Skarnes WC, Pellegrino E, McDonough JA. Improving homology-directed repair efficiency in human stem cells. *Methods*. 2019;**164–165**:18–28. doi: 10.1016/j.ymeth.2019.06.016.

11. Guo Q, Mintier G, Ma-Edmonds M, Storton D, Wang X, Xiao X, et al. “Cold shock” increases the frequency of homology directed repair gene editing in induced pluripotent stem cells. *Sci Rep*. 2018;**8**:2080. doi: 10.1038/s41598-018-20358-5.

12. Jumper J, Evans R, Pritzel A, Green T, Figurnov M, Ronneberger O, Tunyasuvunakool K, Bates R, Žídek A, Potapenko A, et al. 2021. Highly accurate protein structure prediction with AlphaFold. *Nature* **596**: 583–589.

13. Case DA, Ben-Shalom IY, Brozell SR, Cerutti DS, Cheatham III TE, Cruzeiro VWD, et al. Amber 2018. San Francisco: University of California; 2018.

14. Alfieri C, Tischer T, Barford D. A unique binding mode of Nek2A to the APC/C allows its ubiquitination during prometaphase. *EMBO Rep*. 2020;**21**:e49831. doi: 10.15252/embr.201949831.

15. Suijkerbuijk SJE, Van Osch MHJ, Bos FL, Hanks S, Rahman N, Kops GJPL. Molecular causes for BUBR1 dysfunction in the human cancer predisposition syndrome mosaic variegated aneuploidy. *Cancer Res*. 2010;**70**:4891–4900. doi: 10.1158/0008-5472.CAN-09-4319.

16. Homeyer N, Horn AH, Lanig H, Sticht H. AMBER force-field parameters for phosphorylated amino acids in different protonation states: phosphoserine, phosphothreonine, phosphotyrosine, and phosphohistidine. *J Mol Model*. 2006;**12**:281–289. doi: 10.1007/s00894-005-0028-4.

17. Papamokos G V, Tziatzos G, Papageorgiou DG, Georgatos SD, Politou AS, Kaxiras E. Structural role of RKS motifs in chromatin interactions: a molecular dynamics study of HP1 bound to a variably modified histone tail. *Biophys J*. 2012;**102**:1926–33. doi: 10.1016/j.bpj.2012.03.030.

18. Dolinsky TJ, Nielsen JE, McCammon JA, Baker NA. PDB2PQR: an automated pipeline for the setup of Poisson-Boltzmann electrostatics calculations. *Nucleic Acids Res*. 2004;**32**:W665–W667. doi: 10.1093/nar/gkh381.

19. Larini L, Mannella R, Leporini D. Langevin stabilization of molecular-dynamics simulations of polymers by means of quasisymplectic algorithms. *J Chem Phys*. 2007;**126**:104101. doi: 10.1063/1.2464095.

20. Ryckaert JP, Ciccotti G, Berendsen HJC. Numerical integration of the cartesian equations of motion of a system with constraints: molecular dynamics of n-alkanes. *J Comput Phys*. 1977;**23**:327–341.

21. Essmann U, Perera L, Berkowitz ML, Darden T, Lee H, Pedersen LG. A smooth particle mesh Ewald method. *J Chem Phys*. 1998;**103**:8577.

22. Roe D, Cheatham  3rd T. PTRAJ and CPPTRAJ: Software for Processing and Analysis of Molecular Dynamics Trajectory Data. *J Chem Theory Comput*. 2013;**9**:3084–3095. doi: 10.1021/ct400341p.

23. Humphrey W, Dalke A, Schulten K. VMD: visual molecular dynamics. *J Mol Graph*. 1996;**14**:27–28, 33–8. doi: 10.1016/0263-7855(96)00018-5.
