# Supplementary material for: BUB1B monoallelic germline variants contribute to prostate cancer predisposition by triggering chromosomal instability
Source: J Biomed Sci. 2024 Jul 16;31:74. doi: 10.1186/s12929-024-01056-z (PMC11251299; doi:10.1186/s12929-024-01056-z)
Supplement: Supplementary file 2 — Additional file 2. Supplementary Tables (Tables S1-S8). [file 12929_2024_1056_MOESM2_ESM.docx]

# Supplementary Tables

# *BUB1B* monoallelic germline variants contribute to prostate cancer predisposition by triggering chromosomal instability

Maria P. Silva^1,#^, Luísa T. Ferreira^1,#^, Natércia F. Brás^2^, Lurdes Torres^1,3^, Andreia Brandão^1^, Manuela Pinheiro^1^, Marta Cardoso^1^, Adriana Resende^1,3^, Joana Vieira^1,3^, Carlos Palmeira^4^, Gabriela Martins^4^, Miguel Silva^1,3^, Carla Pinto^1,3^, Ana Peixoto^1,3^, João Silva^1,3^, Rui Henrique^5^, Sofia Maia^1^, Helder Maiato^6,7,8^, Manuel R. Teixeira^1,3,9,&^, Paula Paulo^1,&;^*

^1^Cancer Genetics Group, IPO Porto Research Center (CI-IPOP) / RISE@CI-IPOP (Health Research Network), Portuguese Oncology Institute of Porto (IPO Porto) / Porto Comprehensive Cancer Center, Porto, Portugal;

^2^LAQV, REQUIMTE, Department of Chemistry and Biochemistry, Faculty of Sciences, University of Porto, Porto, Portugal;

^3^Department of Laboratory Genetics, Portuguese Oncology Institute of Porto (IPO Porto) / Porto Comprehensive Cancer Center, Porto, Portugal;

^4^Department of Immunology, Portuguese Oncology Institute of Porto (IPO Porto) / Porto Comprehensive Cancer Center, Porto, Portugal;

^5^Department of Pathology, Portuguese Oncology Institute of Porto (IPO Porto) / Porto Comprehensive Cancer Center, Porto, Portugal;

^6^Chromosome Instability & Dynamics Group, Instituto de Investigação e Inovação em Saúde, University of Porto / Porto Comprehensive Cancer Center, i3S, Porto, Portugal;

^7^Cell Division Group, Experimental Biology Unit, Department of Biomedicine, Faculty of Medicine, University of Porto, Porto, Portugal;

^8^Instituto de Biologia Molecular e Celular, University of Porto, Porto, Portugal;

^9^School of Medicine and Biomedical Sciences (ICBAS), University of Porto, Porto, Portugal.

^#,&^ Equal contributions

*Corresponding author, Paula Paulo

**Email:**  paula.paulo@ipoporto.min-saude.pt

| **Table S1.** **Demographic and clinicopathological characteristics of the HPC patients carrying *BUB1B* germline variants.** | | | | | | | |
| --- | --- | --- | --- | --- | --- | --- | --- |
| **Patient ID** | **Age**^a^ | **PSA**^a^ **(ng/ml)** | **Gleason score** | **TNM stage** | **Treatment scheme**^a^ | **Nr. PrCa in the family^b^** | **Nr. Cancers in the family^b^** |
| HPC63 | 49 | 16.03 | 6(3+3) | pT2cNxMx | RP+RT | 1 | 3 |
| HPC119 | 55 | 10.51 | 6(3+3)^c^ | cT3aN0M0 | RT+ADT | 6 | 6 |
| HPC154 | 53 | 4.0 | 7(3+4) | pT2aNxM0 | RP+RT | 2 | 6 |
| HPC227 | 57 | 10.64 | 8(4+4) | pT2cNxM0 | RP+RT+ADT | 2 | 3 |
| HPC262 | 52 | 331 | 7(3+4)^c^ | cT4N0M1 | ADT+CT | 3 | 4 |
| HPC278 | 62 | 17.37 | 8(4+4) | pT3aNxM0 | RP+RT | 2 | 4 |
| HPC343 | 47 | 17.7 | 7(4+3) | pT3bN1M0 | RP+RT | 1 | 2 |
| HPC369 | 54 | 7.22 | 4(2+2) | pT2NxMx | RP | 1 | 1 |
| HPC450 | 61 | 10.0 | 7(3+4)^c^ | cT2cN0M0 | RT+ADT | 2 | 3 |
| ^a^ at/upon diagnosis; ^b^ Family history considering both maternal and paternal lineages; ^c^ Biopsy is the source of the Gleason score. RP- Radical prostatectomy; ADT- Androgen deprivation therapy; RT- Radiotherapy; CT- Chemotherapy. | | | | | | | |

| **Table S2.** **Sequences of the primers/oligos used in the different methods.** | |
| --- | --- |
| **Primer name/application** | **5’🡪3’ Sequence** |
| *KASP Genotyping* |  |
| c.1171_1173 - Forward | AAGAAGGAGATCCTCTACAAAGGG |
| c.1171_1173 - Reverse Wt specific | GAAGGTGACCAAGTTCATGCTAATACATCATCTTCTCTTTCTTCTCCTC |
| c.1171_1173 - Reverse Mut specific | GAAGGTCGGAGTCAACGGATTAATACATCATCTTCTCTTTCTTCTCA |
| *Conventional PCR and Sanger sequencing* |  |
| Exon 9 *-* Forward | GAACATAATTATTGATGGCCCTTGT |
| Exon 9 *-* Reverse | AGGTCAGCTCAACCTTCACA |
| *qRT-PCR* |  |
| Exon 2 *-* Forward | GGGAGATGAATGGGAACTGA |
| Exon 3 *-* Reverse | CCAAACATCCAGAGGGTCAT |
| Exon 2/3 TaqMan Probe (5’FAM, 3’TAMRA) | ATTCAAATGCCCGTTTCTGCTGCTG |
| *Droplet digital PCR (ddPCR)* |  |
| Exon 9 *-* Forward | AGCACCAGAAAGCCTGGAAA |
| Exon 10 *-* Reverse | CTCTGCACTGGTCAATAGCT |
| c.1171_1173del TaqMan Probe (5’FAM, 3’VIC) | TCTTTCTTCTCAGACGCTTGCTGATGG |
| c.1133_1156del TaqMan Probe (5’FAM, 3’VIC) | ATTCAAATGCCCGTTTCTGCTGCTG |
| *CRISPR/Cas9* |  |
| sgRNA1 | TGGCTCTGAACCCTTTGTAG |
| sgRNA2 | AGAGAAGATGATGTATTGTA |
| CRISPR/Cas9 HDR donor (c.1171_1173del) | |
| AAGGAGAATTCCCCTACTCCTGCATAAATCTTCTCTTTACAATACATCATCTTCTCTTTCTTCTCAGACGCTTGCTGATGGCTCTGAACCCTTTGTAGAGGATCTCCTTCTTCCTTTCCAGGCTTTCTGGTGCTT | |
| Note: Tails in KASP primers are underlined (not gene specific). | |

| **Table S3.** **Microsatellite markers of the seven patients, and available relatives, carrying the *BUB1B* variant c.1171_1173del** | | | | | | | | | | | | |
| --- | --- | --- | --- | --- | --- | --- | --- | --- | --- | --- | --- | --- |
| **Index patient/relative** | | **Microsatellite Markers** | | | | | | | | | | |
|  |  | **D15S118** | **D15S1012** | **D15S1044** | **D15S146** | **D15S214** | ***BUB1B^#^*** | **DTR20GT** | **D15S968** | **AFM196XB8** | **D15S781** |  |
| HPC63 | Index* | 217/**221** | **153**/153 | **193**/205 | **206/210** | **267**/**267** | **+**/- | **157/159** | **142/144** | 223/**233** | 182/**184** |  |
|  | son* | **221**/221 | **153**/159 | **193**/193 | **206/210** | 261/**267** | **+**/- | **157/159** | **142/144** | **223**/227 | **184**/184 |  |
| HPC119 | Index* | 221/221 | 153/159 | 193/193 | 206/210 | **267**/**267** | **+**/- | 137/161 | 134/142 | 221/225 | 182/184 |  |
| HPC154 | Index* | 213/225 | 157/163 | 199/203 | 208/208 | 261/**267** | **+**/- | 137/159 | 140/146 | 211/225 | 182/184 |  |
| HPC227 | Index | 213/221 | 159/159 | 199/201 | 208/210 | 263/**267** | **+**/- | 163/163 | 144/146 | 221/225 | 184/186 |  |
| HPC262 | Index* | **215/**221 | **163**/163 | 195/**203** | **206**/206 | **267**/267 | **+**/- | **137**/163 | **138**/142 | **221**/225 | **184**/184 |  |
|  | Daughter | **215**/217 | **163**/169 | 193/**203** | **206**/208 | 265/**267** | **+**/- | **137**/139 | **138**/142 | 219/**221** | 182/**184** |  |
|  | Son* | **215**/221 | **163**/163 | 193/**203** | **206/**208 | **267**/267 | **+**/- | **137**/163 | 134/**138** | **221**/227 | **184**/184 |  |
| GaCa | Index | 215/**221** | **157**/163 | **201**/201 | **208**/210 | 267/**267** | **+**/- | **159**/159 | **140**/140 | **225**/225 | 182/**184** |  |
|  | Father* | 213/**221** | **157**/159 | 193/**201** | 204/**208** | 261/**267** | **+**/- | 137/**159** | 130/**140** | **225**/225 | 182/**184** |  |
|  | Mother* | 215/225 | 153/163 | 201/203 | 206/210 | 261/267 | -/- | 159/161 | 140/142 | 225/225 | 182/182 |  |
|  | Brother* | 213/215 | 159/163 | 193/201 | 204/210 | 261/267 | -/- | 137/159 | 130/140 | 225/225 | 182/182 |  |
|  | Sister | 213/225 | 153/159 | 193/203 | 204/206 | 261/261 | -/- | 137/161 | 130/142 | 225/225 | 182/182 |  |
| LuCa | Index | 221/225 | 153/157 | 201/201 | 204/208 | 261/**267** | **+**/- | 161/161 | 140/144 | 211/223 | 184/186 |  |
| * PCS analysis performed (Table S6); ^#^Genotype for the *BUB1B* variant c.1171_1173del; Conserved region is marked in shadowed grey. Possible shared haplotype in each family is marked in bold letters. GaCa- Gastric Cancer; LuCa- Lung Cancer. | | | | | | | | | | | |  |

| **Table S4. Variation of the SASA values of key BubR1 regions in mutant proteins relatively to WT.** | | | | | | | |
| --- | --- | --- | --- | --- | --- | --- | --- |
| **Variant** | **KEN-1** | **KEN-2** | **TPR3**  (150-185) | **Loop**  (368-379) | **Bub1**  (440-460) | **PK**  (766-1050) | **Full protein** |
| BubR1^WT^ | 0 ± 46 | 0 ± 59 | 0 ± 103 | 0 ± 64 | 0 ± 145 | 0 ± 492 | 0 ± 1016 |
| BubR1^R120Q^ | -62 ± 47 | **-116 ± 58** | -79 ± 100 | 50 ± 82 | -159 ± 139 | -499 ± 436 | 607 ± 928 |
| BubR1^I147T^ | -28 ± 38 | -98 ± 53 | -173 ± 112 | **285 ± 76** | -80 ± 156 | 316 ± 440 | -52 ± 1017 |
| BubR1^R244C^ | 15 ± 38 | 34 ± 55 | **-451 ± 114** | -31 ± 76 | -140 ± 137 | -584 ± 433 | **-3201 ± 2523** |
| BubR1^Δ391^ | **-166 ± 37** | 51 ± 56 | **-270 ± 104** | 94 ± 73 | -182 ± 128 | -519 ± 414 | **-2935 ± 1084** |
| BubR1^R416Q^ | **-146 ± 39** | 1 ± 57 | -37 ± 112 | **328 ± 90** | -58 ± 136 | 613 ± 450 | 238 ± 1196 |
| BubR1^F175G^ | 20 ± 40 | **-147 ± 45** | **-238 ± 103** | 142 ± 62 | -210 ± 125 | 339 ± 518 | -1978 ± 1033 |
| BubR1^F175L^ | **-166 ± 48** | -8 ± 53 | **-351 ± 113** | 93 ± 80 | **-459 ± 143** | 265 ± 475 | -754 ± 1373 |
| BubR1^E413K^ | **-104 ± 40** | -96 ± 50 | -168 ± 105 | **183 ± 89** | 311 ± 206 | -534 ± 462 | **-3099 ± 1358** |
| SASA: solvent accessible surface area;  Notes: SASA values are in Å^3^. Statistically different values to BubR1^WT^ are highlighted in bold. | | | | | | | |

| **Table S5.** **Distance variation between the main helical structures of the TPR in mutant proteins relatively to WT.** | | | | | | | |
| --- | --- | --- | --- | --- | --- | --- | --- |
| **Variant** | **TPR1-A to TPR1-B** | **TPR2-A to TPR2-B** | **TPR3-A to TPR3-B** | **C-term-αH to TPR3-A** | **C-term-αH to TPR3-B** | **TPR1 to TPR2** | **TPR2 to TPR3** |
| BubR1^WT^ | 0.0 ± 0.2 | 0.0 ± 0.3 | 0.0 ± 0.2 | 0.0 ± 0.7 | 0.0 ± 0.9 | 0.0 ± 0.3 | 0.0 ± 0.3 |
| BubR1^R120Q^ | 0.0 ± 0.3 | -0.2 ± 0.3 | 0.0 ± 0.2 | **2.0 ± 0.8** | 1.5 ± 0.8 | 0.2 ± 0.4 | -0.1 ± 0.3 |
| BubR1^I147T^ | -0.1 ± 0.2 | 0.3 ± 0.3 | 0.1 ± 0.2 | **3.0 ± 0.7** | 1.2 ± 0.8 | -0.7 ± 0.4 | -0.1 ± 0.3 |
| BubR1^R244C^ | 0.0 ± 0.3 | -0.2 ± 0.3 | 0.1 ± 0.2 | -0.3 ± 1.0 | -0.4 ± 0.8 | -0.4 ± 0.3 | -0.2 ± 0.3 |
| BubR1^Δ391^ | 0.1 ± 0.2 | 0.2 ± 0.3 | 0.1 ± 0.2 | -0.2 ± 0.7 | -0.2 ± 0.8 | 0.0 ± 0.4 | -0.2 ± 0.3 |
| BubR1^R416Q^ | 0.0 ± 0.2 | 0.2 ± 0.3 | 0.1 ± 0.2 | -1.3 ± 0.7 | **-4.9 ± 0.9** | **-0.8 ± 0.3** | -0.4 ± 0.3 |
| BubR1^F175G^ | 0.0 ± 0.2 | -0.1 ± 0.3 | 0.0 ± 0.2 | -0.2 ± 0.6 | **-4.0 ± 0.8** | -0.2 ± 0.3 | -0.1 ± 0.3 |
| BubR1^F175L^ | 0.2 ± 0.3 | 0.3 ± 0.3 | 0.0 ± 0.2 | **-2.1 ± 0.7** | -0.2 ± 0.8 | -0.3 ± 0.4 | -0.7 ± 0.3 |
| BubR1^E413K^ | -0.2 ± 0.2 | 0.0 ± 0.3 | 0.0 ± 0.2 | -0.9 ± 0.7 | **-5.6 ± 1.1** | -0.5 ± 0.3 | -0.5 ± 0.3 |
| Notes: Distance values are in Å. Statistically valid differences are highlighted in bold. | | | | | | | |

| **Table S6. Variation (%) of the secondary structural elements of all BubR1 mutants relatively to the WT.** | | | | | | |
| --- | --- | --- | --- | --- | --- | --- |
| **Region** | **Variant** | **Secondary structural elements** | | | | |
|  |  | **β-sheet** | **3-10 α-helix** | **α-helix** | **Turn** | **Bend** |
| **TPR domain** | **R120Q** | 0.0 | 0.0 | 1.6 | **-2.1** | 0.6 |
|  | **I147T** | 0.0 | -14.7 | **22.4** | -9.9 | 2.2 |
|  | **R244C** | 0.3 | -8.2 | **12.8** | -2.7 | -2.1 |
|  | **Δ391** | 0.0 | -6.5 | **11.5** | -2.3 | -2.6 |
|  | **R416Q** | 0.0 | -4.6 | **4.9** | 1.4 | -1.7 |
|  | **F175G** | 0.0 | **-18.1** | 13.2 | -1.5 | 6.4 |
|  | **F175L** | 0.0 | -7.4 | **9.4** | -2.8 | 0.9 |
|  | **E413K** | 0.0 | -4.6 | **19.3** | -12.4 | -2.3 |
| **KEN-1** | **R120Q** | 0.0 | -2.2 | **-22.0** | 22.0 | 2.3 |
|  | **I147T** | 0.0 | -7.1 | **9.9** | -3.7 | 0.9 |
|  | **R244C** | 0.0 | 13.3 | -2.7 | -9.3 | -1.3 |
|  | **Δ391** | 0.0 | 38.0 | **-44.7** | 8.8 | -2.1 |
|  | **R416Q** | 0.0 | 18.1 | **-45.9** | 22.2 | 5.6 |
|  | **F175G** | 0.0 | -2.6 | -7.2 | **9.5** | 0.4 |
|  | **F175L** | 0.0 | 18.5 | **-22.9** | -12.8 | 17.2 |
|  | **E413K** | 0.0 | -7.5 | -7.4 | **13.9** | 1.1 |
| **KEN-2** | **R120Q** | 0.0 | -8.8 | -17.6 | 8.5 | **17.9** |
|  | **I147T** | 0.0 | 4.8 | **-33.1** | 25.9 | 2.4 |
|  | **R244C** | 0.0 | **-38.4** | 6.1 | 3.7 | 28.6 |
|  | **Δ391** | 0.0 | **-31.5** | 17.8 | 12.1 | 1.5 |
|  | **R416Q** | 0.0 | -24.4 | -33.1 | **37.8** | 19.7 |
|  | **F175G** | 0.2 | -46.6 | -15.1 | **62.0** | -0.5 |
|  | **F175L** | 0.0 | -4.8 | -23.4 | **24.0** | 4.2 |
|  | **E413K** | 0.0 | **-38.4** | 6.1 | 3.7 | 28.6 |
| **Loop** | **R120Q** | 1.4 | -6.1 | 13.0 | **-15.7** | 7.3 |
|  | **I147T** | **-6.2** | -2.0 | -1.7 | 5.4 | 4.4 |
|  | **R244C** | -6.3 | -6.1 | 2.0 | -9.9 | **20.3** |
|  | **Δ391** | -4.0 | -4.8 | 7.9 | -8.5 | **9.4** |
|  | **R416Q** | -2.4 | -1.7 | -4.4 | -1.4 | **9.9** |
|  | **F175G** | -6.3 | -5.9 | 4.6 | -14.8 | **22.4** |
|  | **F175L** | -6.3 | 6.1 | -2.7 | -3.8 | **6.8** |
|  | **E413K** | -6.1 | -4.7 | -5.7 | -8.8 | **25.3** |
| **Full protein** | **R120Q** | -0.3 | 1.4 | **-1.7** | 0.9 | -0.2 |
|  | **I147T** | -0.3 | 0.5 | **-0.8** | 0.6 | 0.1 |
|  | **R244C** | -0.9 | -0.6 | 0.2 | -0.1 | **1.4** |
|  | **Δ391** | -0.9 | -0.2 | 0.1 | -0.2 | **1.2** |
|  | **R416Q** | -0.7 | 0.1 | -0.8 | **0.9** | 0.5 |
|  | **F175G** | -1.0 | -0.7 | 0.0 | **1.3** | 0.1 |
|  | **F175L** | -0.8 | -0.3 | 0.8 | -0.6 | **0.9** |
|  | **E413** | -0.8 | 0.0 | -0.2 | -0.3 | **1.2** |
| Note: Higher differences are highlighted in bold numbers and shown in the main manuscript. The negative and positive mutant controls are colored in green and red, respectively. | | | | | | |

| **Table S7.** **Intramolecular contacts made by key residues of the different BubR1 mutants.** | | | | | | | | | | | |
| --- | --- | --- | --- | --- | --- | --- | --- | --- | --- | --- | --- |
| **Residue 1** | **Residue 2** |  | | **Frequency of interaction (%)** | | | | | | | |
|  |  | **WT** | **R120Q** | | **I147T** | **R244C** | **Δ391** | **R416Q** | **F175G** | **F175L** | **E413K** |
| K26 | W22 | 50 | 37 | | 52 | 30 | - | 45 | 40 | 37 | 38 |
| N28 | S25 | - | - | | - | - | - | 41 | 30 | - | - |
| R120 | D73 | 47 | - | | 74 | 51 | 62 | 65 | 53 | 65 | 56 |
|  | D118 | 63 | - | | 85 | 88 | 93 | 49 | 83 | 63 | 48 |
| I147 | L142 | 40 | 39 | | - | 46 | 37 | 37 | 39 | 35 | - |
|  | Q145 | - | - | | 35 | - | - | - | - | - | - |
| E161 | R33 | 52 | - | | 61 | 34 | 33 | 39 | 31 | 62 | 31 |
| N306 | A303 | 33 | - | | 34 | 36 | - | 37 | 34 | - | - |
| Q386 | V382 | 40 | 32 | | - | 39 | 39 | 40 | - | 33 | - |
|  | E391 | 30 | 30 | | - | 33 | - | - | - | 31 | 34 |
| R416 | F412 | 49 | 58 | | - | 53 | 37 | 33 | 39 | 34 | 57 |
|  | E413 | 30 | 36 | | 30 | - | 62 | - | - | 30 | - |
|  | F420 | - | 35 | | - | - | 38 | - | - | - | - |
| D610 | A614 | 52 | - | | - |  | 43 | - | 55 | - | 45 |
| F611 | A615 | 55 | 43 | | 41 |  | 52 | - | - | 49 | 45 |
| S670 | K668 | 32 | 35 | | - | 73 | - | 61 | 32 | - | 45 |
| R727 | S915 | 74 | - | | 43 | - | - | - | 30 | - | 35 |
|  | Q921 | 52 | - | | - | - | - | - | - | - | - |
|  | L731 | 49 | - | | 45 | 43 | 35 | 50 | 46 | 33 | 44 |
|  | P721 | 45 | 37 | | 43 | 58 | - | - | 38 | - | 37 |
|  | C723 | 43 | 47 | | 57 | 52 | 50 | 53 | 58 | 55 | 50 |
|  | S913 | - | 53 | | 40 | 52 | 64 | 82 | 43 | 53 | 46 |
| D777 | S798 | 34 | - | | - | - | 30 | 34 | - | - | - |
|  | S797 | - | - | | - | 46 | 35 | - | - | 58 | 38 |
| Y778 | D911 | 59 | 43 | | 94 | - | 59 | 67 | 42 | 32 | 30 |
|  | C775 | 57 | 45 | | 33 | 34 | 49 | 48 | - | 48 | 50 |
| K795 | V834 | 66 | 74 | | 89 | 62 | 69 | 75 | 81 | 77 | 69 |
| D882 | S884 | 68 | 50 | | 49 | - | 44 | 49 | 39 | 36 | 50 |
|  | R931 | 58 | 38 | | 31 | 49 | - | 34 | - | - | - |
|  | R886 | 50 | - | | 31 | - | 31 | - | 46 | 58 | 33 |
| Note: Only the H-bonds with frequency higher than 30% along the simulations were considered. | | | | | | | | | | | |

| Table S8. Premature chromatid separation (PCS) frequencies of healthy controls and HPC carriers of *BUB1B* variants. | | | | |
| --- | --- | --- | --- | --- |
| Sample | **Age^a^** | **cDNA change** | **Protein change** | **PCS (%)** |
| Control 1 | 50 | -/- | -/- | 0.75% |
| Control 2 | 51 | -/- | -/- | 1.36% |
| Control 3 | 51 | -/- | -/- | 2.41% |
| Control 4 | 52 | -/- | -/- | 2.57% |
| Control 5 | 45 | -/- | -/- | 4.29% |
| Control 6 | 67 | -/- | -/- | 4.41% |
| Control 7 | 42 | -/- | -/- | 5.67% |
| Control 8 | 78 | -/- | -/- | 8.64% |
| Control 9 | 45 | -/- | -/- | 8.72% |
| Control 10 | 45 | -/- | -/- | 8.74% |
| HPC63 son (healthy) | 40 | c.1171_1173del | p.(Glu391del) | 19% |
| HPC262 son (healthy) | 25 | c.1171_1173del | p.(Glu391del) | 70% |
| HPC63 | 67 | c.1171_1173del | p.(Glu391del) | 34% |
| HPC119 | 72 | c.1171_1173del | p.(Glu391del) | 37% |
| HPC154 | 72 | c.1171_1173del | p.(Glu391del) | 34% |
| HPC262 | 67 | c.1171_1173del | p.(Glu391del) | 54% |
| HPC278 | 72 | c.2481del | p.(Gln827HisfsTer13) | 28% |
| HPC450 | 72 | c.359G>A | p.(Arg120Gln) | 61% |
| ^a^ at the time of blood collection. -/-, negative genotype for the variant c.1171_1173del; p.(Glu391del). | | | | |
|  | | | | |
