# Supplementary material for: BUB1B monoallelic germline variants contribute to prostate cancer predisposition by triggering chromosomal instability
Source: J Biomed Sci. 2024 Jul 16;31:74. doi: 10.1186/s12929-024-01056-z (PMC11251299; doi:10.1186/s12929-024-01056-z)
Supplement: Supplementary file 3 — Additional file 3. Supplementary Figures (Figures S1-S5). [file 12929_2024_1056_MOESM3_ESM.docx]

# Supplementary Figures

# *BUB1B* monoallelic germline variants contribute to prostate cancer predisposition by triggering chromosomal instability

Maria P. Silva^1,#^, Luísa T. Ferreira^1,#^, Natércia F. Brás^2^, Lurdes Torres^1,3^, Andreia Brandão^1^, Manuela Pinheiro^1^, Marta Cardoso^1^, Adriana Resende^1,3^, Joana Vieira^1,3^, Carlos Palmeira^4^, Gabriela Martins^4^, Miguel Silva^1,3^, Carla Pinto^1,3^, Ana Peixoto^1,3^, João Silva^1,3^, Rui Henrique^5^, Sofia Maia^1^, Helder Maiato^6,7,8^, Manuel R. Teixeira^1,3,9,&^, Paula Paulo^1,&;^*

^1^Cancer Genetics Group, IPO Porto Research Center (CI-IPOP) / RISE@CI-IPOP (Health Research Network), Portuguese Oncology Institute of Porto (IPO Porto) / Porto Comprehensive Cancer Center, Porto, Portugal;

^2^LAQV, REQUIMTE, Department of Chemistry and Biochemistry, Faculty of Sciences, University of Porto, Porto, Portugal;

^3^Department of Laboratory Genetics, Portuguese Oncology Institute of Porto (IPO Porto) / Porto Comprehensive Cancer Center, Porto, Portugal;

^4^Department of Immunology, Portuguese Oncology Institute of Porto (IPO Porto) / Porto Comprehensive Cancer Center, Porto, Portugal;

^5^Department of Pathology, Portuguese Oncology Institute of Porto (IPO Porto) / Porto Comprehensive Cancer Center, Porto, Portugal;

^6^Chromosome Instability & Dynamics Group, Instituto de Investigação e Inovação em Saúde, University of Porto / Porto Comprehensive Cancer Center, i3S, Porto, Portugal;

^7^Cell Division Group, Experimental Biology Unit, Department of Biomedicine, Faculty of Medicine, University of Porto, Porto, Portugal;

^8^Instituto de Biologia Molecular e Celular, University of Porto, Porto, Portugal;

^9^School of Medicine and Biomedical Sciences (ICBAS), University of Porto, Porto, Portugal.

^#,&^ Equal contributions

*Corresponding author, Paula Paulo

**Email:**  [paula.paulo@ipoporto.min-saude.pt](mailto:paula.paulo@ipoporto.min-saude.pt)


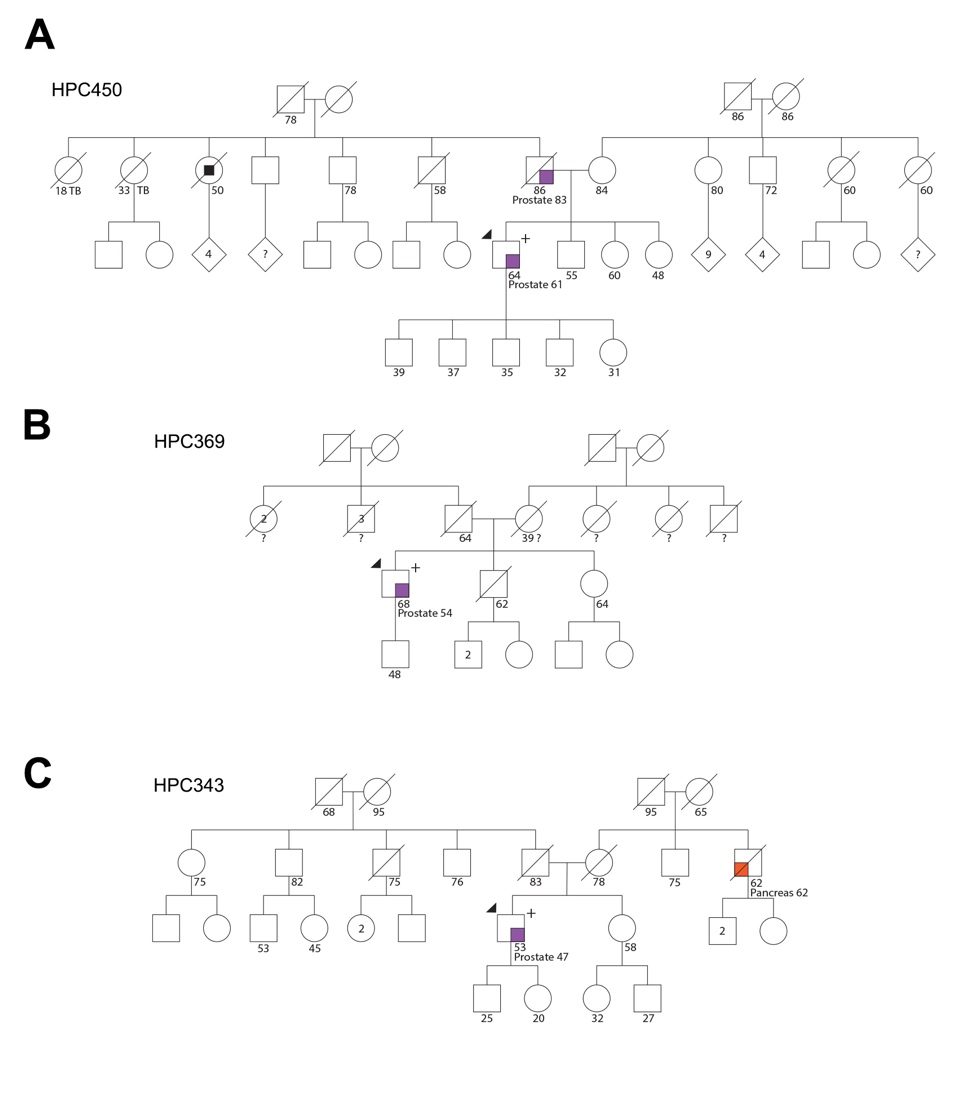


**Figure S1. Pedigrees of the HPC cases carrying missense *BUB1B* variants. A)** Carrier of the *BUB1B* missense variant c.359G>A; p.(Arg120Gln). **B)** Carrier of the *BUB1B* missense variant c.440T>C; p.(Ile147Thr). **C)** Carrier of the *BUB1B* missense variant c.1247G>A; p.(Arg416Gln). Carrier patients are labeled with a “plus” mark. Squares represent the males, circles the females and diamonds unknown gender. Deceased individuals are represented by a diagonal line through a symbol and the affected ones are highlighted by colored symbols. The index case is indicated by an upper left arrow and the cancer type and age at diagnosis are indicated whenever known.


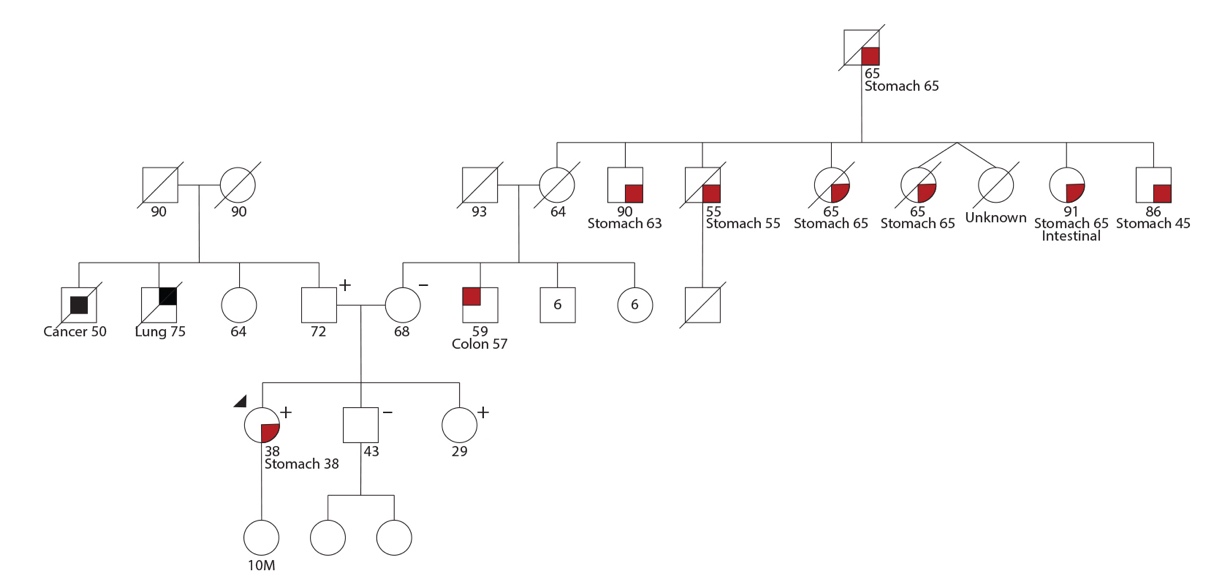


**Figure S2. Pedigree of the gastric cancer patient with phased haplotype carrying the in-frame variant c.1171_1173del.**


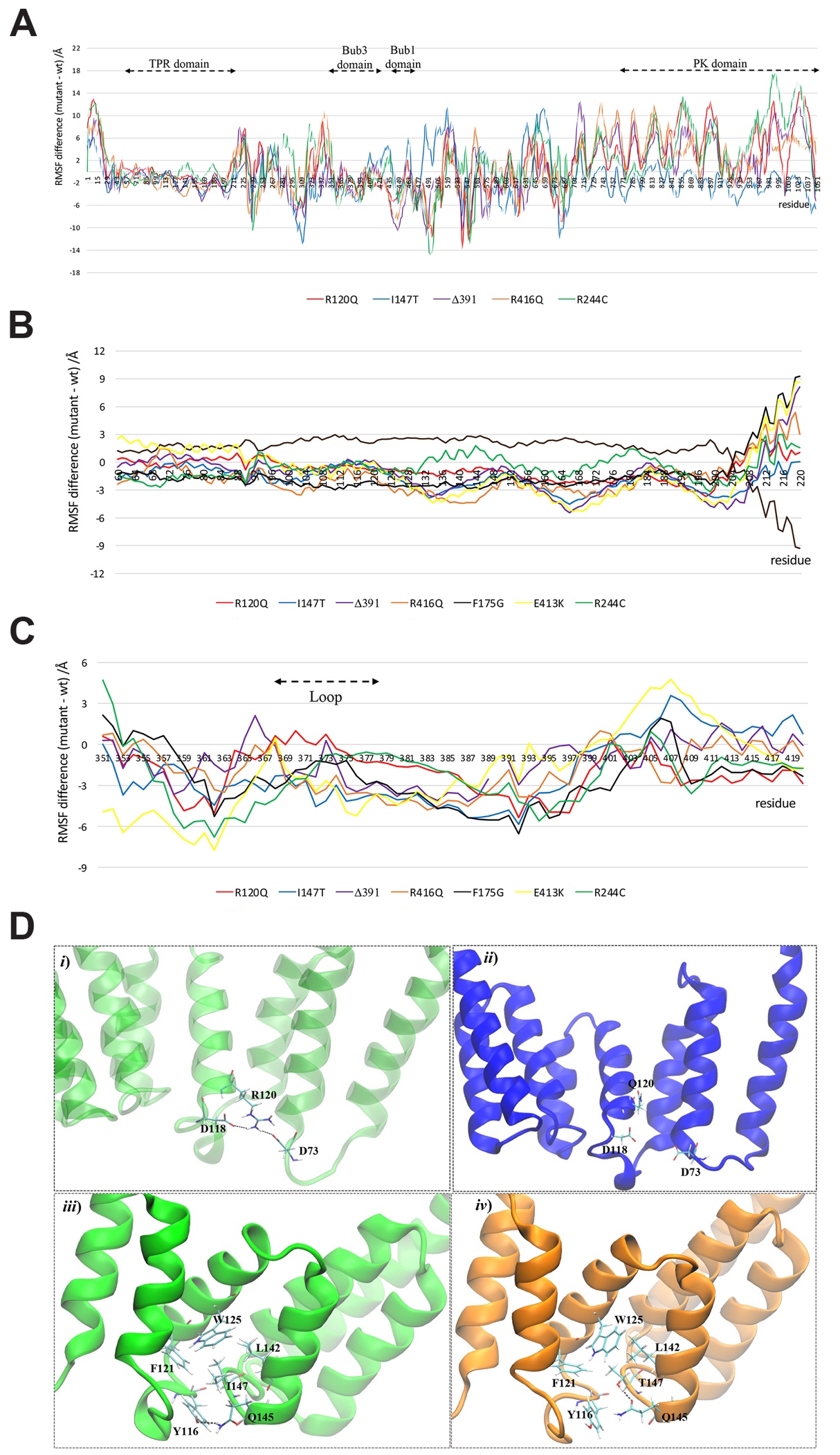


**Figure S3. MD simulations highlight structural differences between BubR1 mutants and WT proteins.** MD simulations for **A)** complete BubR1 protein, **B)** BubR1^TPR^ (60 – 220 residues) and **C)** BubR1^Bub3^ (351 – 420 residues) showing variations in RMSF values. **D)** representative MD structures of the TPR region for the BubR1^WT^ (green, *i* and *iii*), BubR1^R120Q^ (blue, *ii*) and BubR1^I147T^ (orange, *iv*). Proteins are depicted as cartoon, while the residues are represented in sticks and colored by atom type. Some key distances are also indicated.


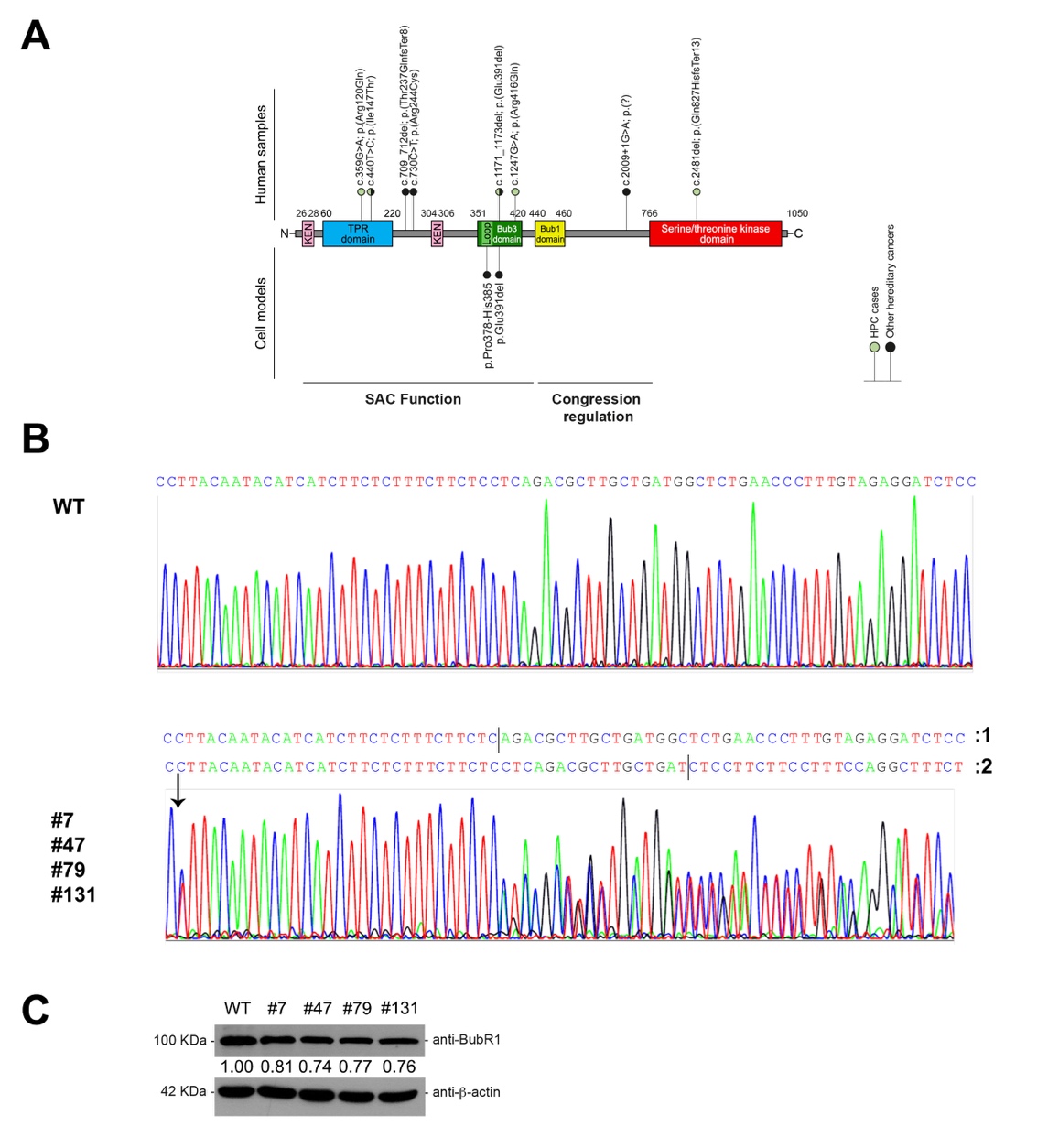


**Figure S4. *BUB1B* gene-edited cell clones used in phenotypic assays. A)** Schematical representation of the BubR1 protein showing the location of the variants identified in patients and in the *in vitro* cell models used in this study. **B)** Representative Sanger sequencing electropherograms for the WT population and the *BUB1B*-edited clones containing the c.1171_1173del variant (read **:1**). The four clones (#7, #47, #79 and #131) revealed to, additionally, harbour the variant c.1133_1156del (read **:2**). An arrow highlights the synonymous variant included in the PAM sequence of the HDR template to avoid cleavage by Cas9. Deletion sites are shown by a vertical line in each sequence. **C)** BubR1 expression levels in all clonal populations comparing with WT cells, assessed by western-blot. Relative BubR1 expression in each cell population were obtained by correcting to its respective β-actin levels and normalized to WT. Clones #47 and #131 were used in all experiments and named C1 and C2, respectively, for simplification. In the chase experiments, clone #79 (named C3) was also used.


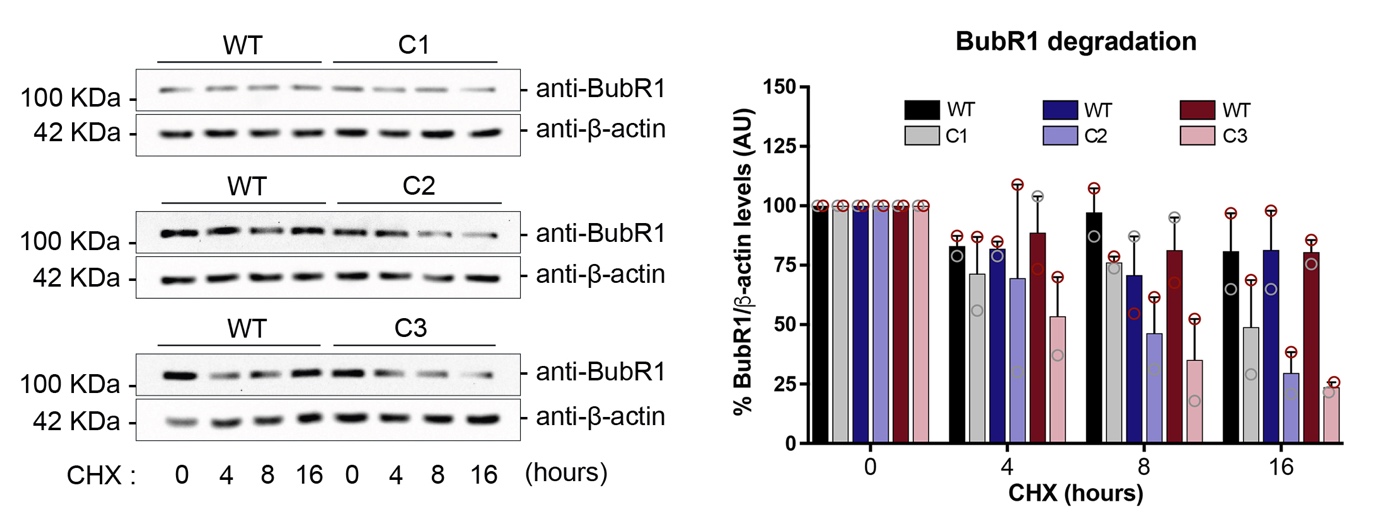


**Figure S5. Analysis of BubR1 expression upon cycloheximide (CHX) treatment in WT and *BUB1B* gene-edited cells.** Representative images from western blot analysis of BubR1 expression before (0h) and after 4h, 8h and 16h of treatment with CHX are shown at *left*. Blots for C1 and C2 clones are the same shown in the main manuscript, here used for comparison with data obtained for a 3^rd^ independent clone (named C3) harboring the same genotype. Densitometry analysis of western blot images is shown at *right*. For every time point, BubR1 expression levels are shown for each WT-paired clonal cell population (C1, C2 and C3). The % of BubR1 expression was obtained by normalizing BubR1 levels to those of the corresponding β-actin and adjusting to the expression levels obtained before CHX treatment (at 0h). For comparison, different colored circles highlight matched clone/WT pairs from the same assay.
